# Saturation transfer properties of tumour xenografts derived from prostate cancer cell lines 22Rv1 and DU145

**DOI:** 10.1038/s41598-020-78353-8

**Published:** 2020-12-04

**Authors:** Ziyu Tan, Wilfred W. Lam, Wendy Oakden, Leedan Murray, Margaret M. Koletar, Stanley K. Liu, Greg J. Stanisz

**Affiliations:** 1 Physical Sciences, Sunnybrook Research Institute, Toronto, ON Canada; 2 Medical Biophysics, University of Toronto, Toronto, ON Canada; 3 Radiation Oncology, University of Toronto, Toronto, ON Canada; 4 Biological Sciences, Sunnybrook Research Institute, Toronto, ON Canada; 5 Radiation Oncology, Sunnybrook Health Sciences Centre, Toronto, ON Canada; 6 Neurosurgery and Paediatric Neurosurgery, Medical University of Lublin, Lublin, Poland

**Keywords:** Cancer imaging, Cancer metabolism, Cancer microenvironment, Tumour biomarkers, Cancer, Health occupations, Medical research, Oncology

## Abstract

Histopathology is currently the most reliable tool in assessing the aggressiveness and prognosis of solid tumours. However, developing non-invasive modalities for tumour evaluation remains crucial due to the side effects and complications caused by biopsy procedures. In this study, saturation transfer MRI was used to investigate the microstructural and metabolic properties of tumour xenografts in mice derived from the prostate cancer cell lines 22Rv1 and DU145, which express different aggressiveness. The magnetization transfer (MT) and chemical exchange saturation transfer (CEST) effects, which are associated with the microstructural and metabolic properties in biological tissue, respectively, were analyzed quantitatively and compared amongst different tumour types and regions. Histopathological staining was performed as a reference. Higher cellular density and metabolism expressed in more aggressive tumours (22Rv1) were associated with larger MT and CEST effects. High collagen content in the necrotic regions might explain their higher MT effects compared to tumour regions.

## Introduction

Prostate cancer (PCa) is the most common non-skin cancer amongst the male population in developed countries^1^. Each year, approximately 1.3 million new PCa cases are diagnosed and nearly 366,000 men die of PCa globally^2^. It is estimated that ~ 30% men over 50 years old show histological evidence of PCa^3^. The most common treatments for localized prostate tumours are radical prostatectomy and prostate radiation therapy^1^, both of which require accurate assessment of the tumour in advance, including evaluation of its aggressiveness through clinicopathological features. Clinically, the tumour assessment workflow involves prostate biopsy. However, complications of this invasive procedure, including hematuria, rectal bleeding, infection, and pain are often reported^4^, which can lead to significantly increased hospital admission rates for complications, with sepsis being potentially life-threatening^5^. Therefore, developing new tumour assessment techniques that are solely based on non-invasive modalities is essential to improved patient management. Previous clinical studies have revealed that tumour necrosis is an important factor in evaluating tumour stage, aggressiveness, prognosis, as well as predicting treatment effectiveness in different cancer types^6–9^.

Currently, the evaluation of tumour necrosis is evaluated through histopathology, yet only a few studies have managed to assess tumour necrosis exclusively with non-invasive modalities. Lang et al.^9^ have applied diffusion MRI measurements to detect necrotic regions in tumours and associated the signal decrease in these areas with low cell integrity. Standish et al.^10^ have used Doppler optical coherence tomography to monitor and quantify the tumour necrosis induced by treatment in PCa. Uhl et al.^11^ reported changes in tumour apparent diffusion coefficient that are related to the degree of tumour necrosis induced by treatment. Jeong et al.^12^ showed that gadolinium mesoporphyrin can enhance tumour necrotic regions, but with very poor sensitivity. Among these modalities, diffusion MRI has a poor signal-to-noise-ratio (SNR) and is sensitive to motion that lead to artifacts, while contrast-enhanced MRI tends to have low sensitivity due to poor penetration of paramagnetic contrast agent^13^. Moreover, most of these modalities focus only on detecting necrotic regions and differentiating them from the active tumour areas without further analyses regarding their intratumoural microstructure and metabolic properties, thus, rendering them insufficient for biological or clinical interpretation.

Saturation transfer is a novel MRI technique that detects the water signal loss caused by magnetization exchange between certain chemical groups or macromolecules and free water molecules. Magnetization transfer (MT)^14^, chemical exchange saturation transfer (CEST)^15^, and the relayed Nuclear Overhauser Effect (rNOE)^16^ are the three major sub-categories of saturation transfer. The MT effect is due to the exchange of magnetization between semisolid macromolecules and water. Macromolecules exist abundantly in biological tissues, e.g., lipids are the main component of the cell and organelle membranes and the extracellular matrix and fibrous tissues have high collagen content. Both collagen and lipid bilayers are thought to be major contributors to MT^17^. The CEST effect is caused by the chemical exchange of protons between chemical groups (e.g., amide and guanidinium) in dissolved proteins and free water molecules. rNOE is a process of intramolecular exchange of magnetization between certain chemical groups (e.g., aliphatic) and those in another chemical group (e.g., hydroxyl^18^), whose protons then undergo chemical exchange with those of free water. Thus, CEST informs about the degree of tissue metabolism. A combination of MT, CEST, and rNOE saturation transfer can provide valuable insights into the microstructural and metabolic properties of tissue without any injection of contrast agent^19,20^.

A saturation transfer experiment starts with a long radiofrequency saturation pulse with amplitude B_1_, during which the longitudinal magnetization of protons resonating at the frequency of the pulse (and related to the chemical groups or macromolecule groups to which it is bound) is significantly decreased. The exchange of its magnetization with that of free water leads to the decrease of the water longitudinal magnetization and, hence, signal of free water. The measurement is repeated for various frequencies and amplitudes of the saturation pulse. The signal is conventionally normalized by the saturation-free signal and displayed as a plot as a function of saturation frequency, which is known as a Z-spectrum.

The current study focused on evaluating the saturation transfer properties of tumour xenografts derived from two different PCa cell lines: commonly used DU145 and more aggressive 22Rv1. The MRI properties of both tumour and necrotic regions were analyzed. Their saturation transfer properties were compared to histopathology to provide a better understanding of the microstructural and metabolic properties of prostate tumours.

## Results

Saturation transfer effects were measured in mouse tumour xenografts derived from DU145 (*n* = 34) and 22Rv1 (*n* = 32) PCa cell lines. Z-spectra at high B_1_ (3 and 6 µT) and low B_1_ (0.5 and 2 µT) were collected from tumour and necrotic regions of these two types of tumours. The MT and CEST effects were isolated by fitting to the two-pool MT model^14^ to the MT-sensitive Z-spectra data. The four independent model parameters are the transverse relaxation time, T_2_, of the free pool (*T*_2,F_), the exchange rate of magnetization from the MT to the free pool (*R*_MT_), the equilibrium magnetization of the MT pool relative to the free pool (*M*_0,MT_), and the T_2_ of the MT pool (*T*_2,MT_). Previously, we found through phantom studies that there is a constant negative correlation between R_MT_ and the solute pool size M_0,MT_ (see Supplementary Fig. S3). Therefore, instead of analyzing them separately, their product is used to represent the MT effect. Results are compared between tumours from these cell lines and between the active tumour and necrotic/apoptotic regions.

### Segmentation results

**Figure 1:**
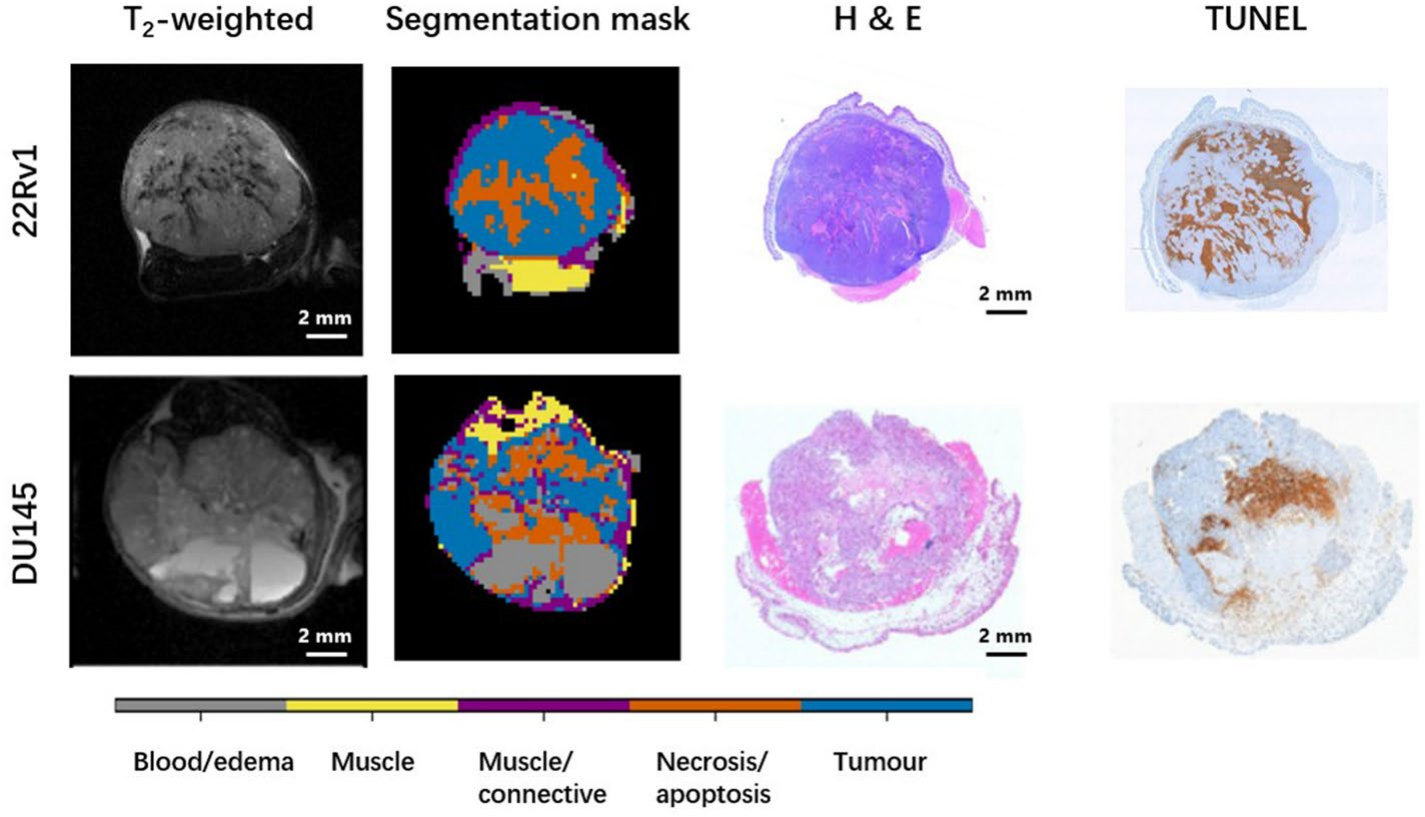
Representative T_2_-weighted anatomical images, segmentation masks, and H&E and TUNEL stains. Similar data containing all the tumours can be found in Supplementary Figs. S1 and S2.

All tumours were automatically segmented into muscle and three intratumoural regions: active tumour (henceforth shortened to tumour), necrotic/apoptotic (shortened to necrotic), and blood/edema using an automatic segmentation algorithm developed by our group^21^. Representative T_2_-weighted anatomical images, segmentation masks, and histopathology stains, which were of consistent quality across mice, are shown in Fig. 1. The masks generated by the segmentation pipeline were used as regions of interest for quantitative analysis of tumour and necrotic regions. A similar figure containing all the tumours can be found in Supplementary Figs. S1 and S2.

### Z-spectrum analysis

**Figure 2:**
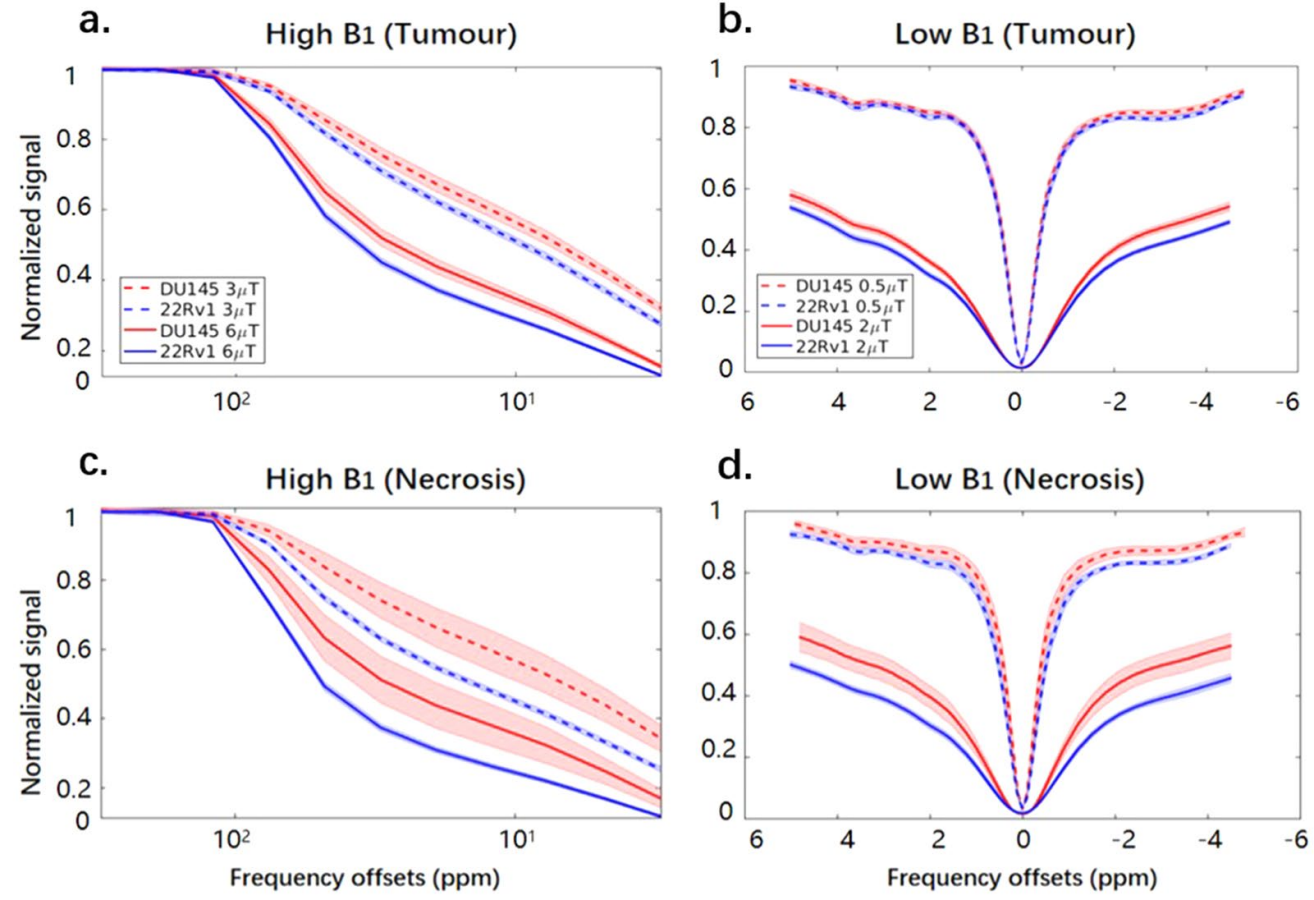
Z-spectra measured from DU145 and 22Rv1 tumour xenografts. Comparisons of average Z-spectra (mean and standard deviation) in (**a**,**b**) tumour and (**c**,**d**) necrotic regions measured with (**a**,**c**) high and (**b**,**d**) low B_1_. Both DU145 tumour and necrotic regions consistently have higher signal (i.e., less saturation transfer contrast) than the corresponding 22Rv1 regions.

Figure 2 shows the ROI-averaged Z-spectra for intratumoural regions in the two tumour types. The saturation transfer effects in the tumour regions are higher (i.e., there is lower signal) in 22Rv1 compared to DU145. This is also seen in the necrotic regions. Both high B_1_s (3 and 6 μT) Z-spectra (Fig. 2a,c) exhibit strong MT effects, which can be observed as a deviation from the sigmoidal curve around 50 ppm. The Z-spectra with the lowest B_1_ (0.5 μT) of both 22Rv1 and DU145 tumour regions (Fig. 2b) have peaks at 3.5, 2.0, and − 3.3 ppm, which are the resonance frequency offsets of the amide, guanidinium, and aliphatic groups, respectively. These peaks are still present with a B_1_ of 2 μT (Fig. 2d), albeit they are more difficult to appreciate because of their increased width. There are also differences between the two types of tumours at lower B_1_ values, where both necrosis and tumour regions of the 22Rv1 tumours (blue lines in Fig. 2b,d) have higher saturation transfer effects than their counterparts in the DU145 tumours (red lines in Fig. 2b,d) at 2 μT (solid lines), but such differences are less pronounced at 0.5 μT (dashed lines).

### Quantitative MT parameters and CEST/rNOE maps

**Figure 3:**
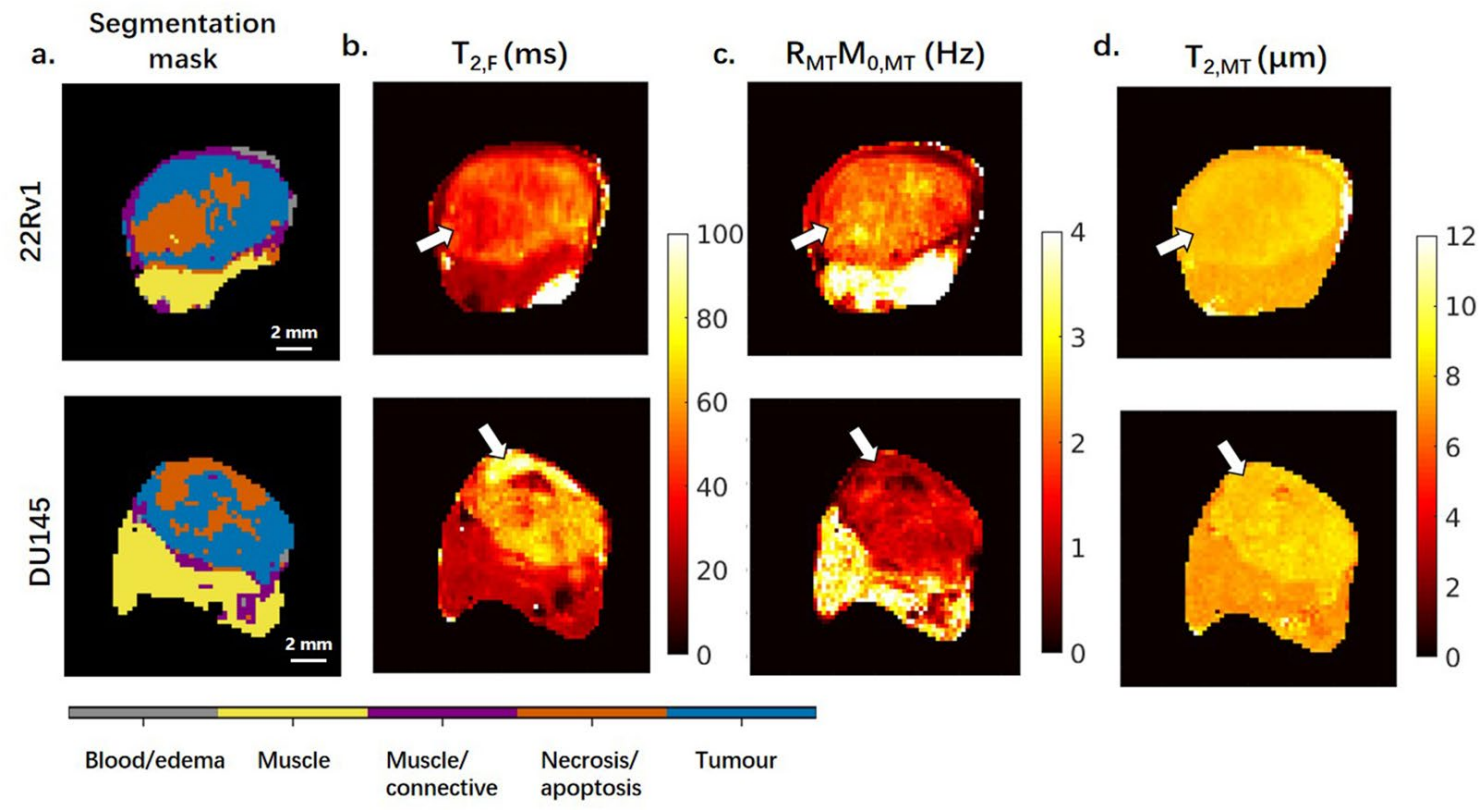
Segmentation masks and maps of the estimated MT model parameters for representative tumours. The model parameters are the transverse relaxation time, T_2_, of the free pool (*T*_2,F_), the exchange rate of magnetization from the MT to the free pool (*R*_MT_), the equilibrium magnetization of the MT pool relative to the free pool (*M*_0,MT_), and the T_2_ of the MT pool (*T*_2,MT_). The product of *R*_MT_ and *M*_0,MT_ is given because they are coupled; together they are a measure of the magnitude of the MT effect.

**Figure 4:**
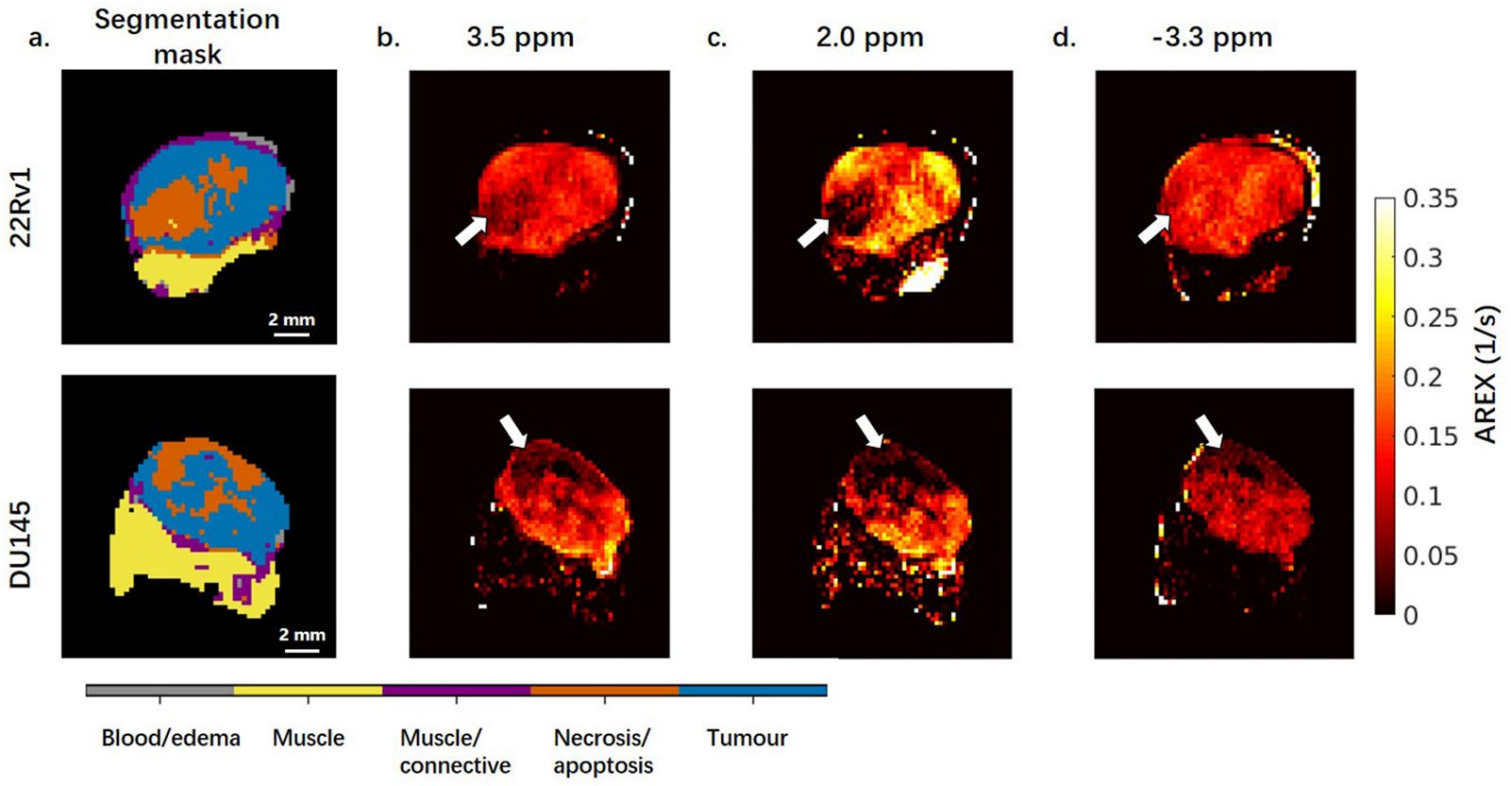
Segmentation masks and maps of the CEST and rNOE contributions at three frequency offsets for representative tumours with a B_1_ of 2 µT. The frequency offsets correspond to the resonances of the (**b**) amide (CEST), (**c**) guanidinium (CEST), and (**d**) aliphatic (rNOE) chemical groups. For both tumours, the active regions of the tumour show higher CEST contributions than the regions of necrosis. The CEST effects (most notably at 2 ppm) are higher in the 22Rv1 tumour. Muscle has negligible CEST effect.

One representative of each type of tumour is shown in Fig. 3 (MT model parameters) and Fig. 4 (CEST/rNOE contributions to the Z-spectra at three frequency offsets). Based on the segmentation of these two tumours in Fig. 3a, the necrotic region (marked with arrows) of the DU145 tumour displays much higher free pool transverse relaxation time, T_2_, (*T*_2,F_; Fig. 3b) than other regions. Both tumour and necrosis (marked with arrows) regions in the 22Rv1 tumour show higher MT effects (*R*_MT_*M*_0,MT_) than their counterparts in DU145 (Fig. 3c). Furthermore, within the 22Rv1 tumour, the MT effect is higher in the necrotic region than the tumour region. High MT effect in the muscle region is also seen in both tumours.

In Fig. 4, the necrotic region (marked with arrows) in the both 22Rv1 and DU145 tumours display much lower CEST effects than their tumour regions at frequency offsets 3.5 and 2.0 ppm. Moreover, the CEST effect for the tumour regions is smaller for the DU145 tumour xenograft.

### Isolating the effects of the free water, MT, CEST, and rNOE pools

**Figure 5:**
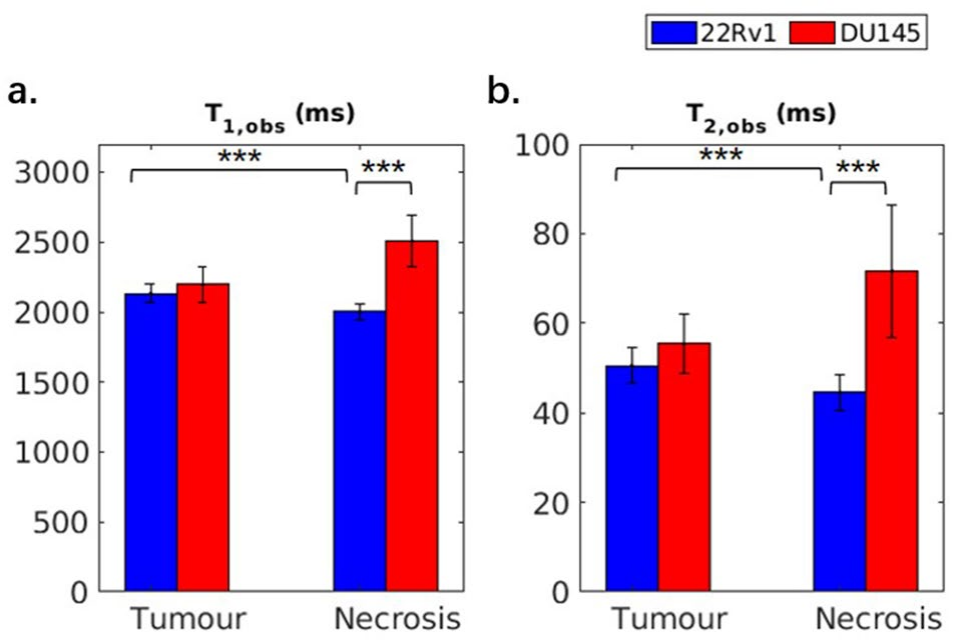
Observed T_1_ and T_2_ of 22Rv1 and DU145 tumour and necrotic regions. There are statistical differences between the tumour and necrotic regions in the 22Rv1 tumours, and between the necrotic regions of 22Rv1 and DU145 tumours. ***p < 0.001.

**Figure 6:**
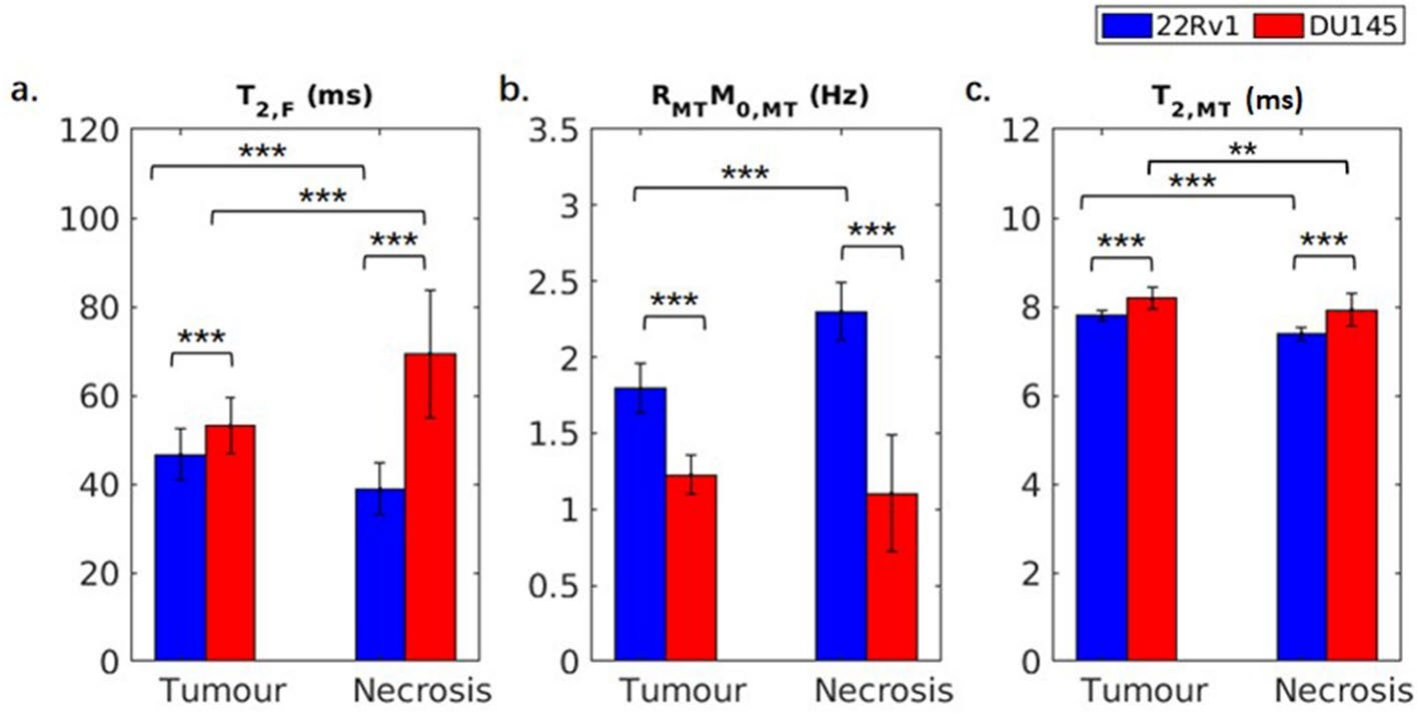
Estimated two-pool MT model parameters of 22Rv1 and DU145 tumour and necrotic regions. The parameters include the transverse relaxation time T_2_ of the free pool (*T*_2,F_), the product of the exchange rate of magnetization from the MT to the free pool (*R*_MT_) and equilibrium magnetization of the MT pool relative to the free pool (*M*_0,MT_), and the T_2_ of the MT pool (*T*_2,MT_). *R*_MT_ and *M*_0,MT_ are multiplied because they are coupled (see Supplementary Fig. S3 for separate comparison); together they represent the magnitude of the MT effect. **p < 0.01. ***p < 0.001.

In Figs. 5 and 6, the observed T_1_ and T_2_ (*T*_1,obs_ and *T*_2,obs_, respectively) and estimated two-pool MT model parameters of tumour and necrotic regions in both tumour types are shown. *T*_1,obs_ and *T*_2,obs_ are both significantly lower in the necrotic region of the 22Rv1 (2000 ± 50 ms and 44 ± 4 ms, respectively) as compared to the DU145 necrotic region (2500 ± 200 ms and 70 ± 20 ms), shown in Fig. 5a. These differences were all significant with *p* < 0.001.

The estimated MT model parameters are shown in Fig. 6. The goodness of fit is shown in Supplementary Fig. S5 and Table S1. The intrinsic transverse relaxation time T_2_ of the free pool (*T*_2,F_) is significantly different between 22Rv1 tumour and necrotic regions (47 ± 6 vs. 39 ± 5 ms) and between 22Rv1 and DU145 necrotic regions (39 ± 5 vs. 69 ± 15 ms). The product of the exchange rate of magnetization from the MT to the free pool (*R*_MT_) and equilibrium MT pool size relative to that of water (*M*_0,MT_), has three significant differences: between 22Rv1 tumour and necrotic regions (1.8 ± 0.2 vs. 2.3 ± 0.6 Hz), between 22Rv1 and DU145 tumour regions (1.8 ± 0.2 vs. 1.2 ± 0.1 Hz), and between 22Rv1 and DU145 necrotic regions (2.3 ± 0.6 vs. 1.1 ± 0.4 Hz) and is shown in Fig. 6b. The intrinsic T_2_ relaxation time of the MT pool (*T*_2,MT_) has four significant differences: between 22Rv1 tumour and necrotic regions (7.8 ± 0.1 vs. 7.4 ± 0.1 µs), between DU145 tumour and necrotic regions (8.2 ± 0.2 vs. 7.9 ± 0.4 µs), between 22Rv1 and DU145 tumour regions (7.8 ± 0.1 vs. 8.2 ± 0.2 µs), and between 22Rv1 and DU145 necrotic regions (7.4 ± 0.1 vs. 7.9 ± 0.4 µs) and is shown in Fig. 6c. These differences were all significant to p < 0.001.

**Figure 7:**
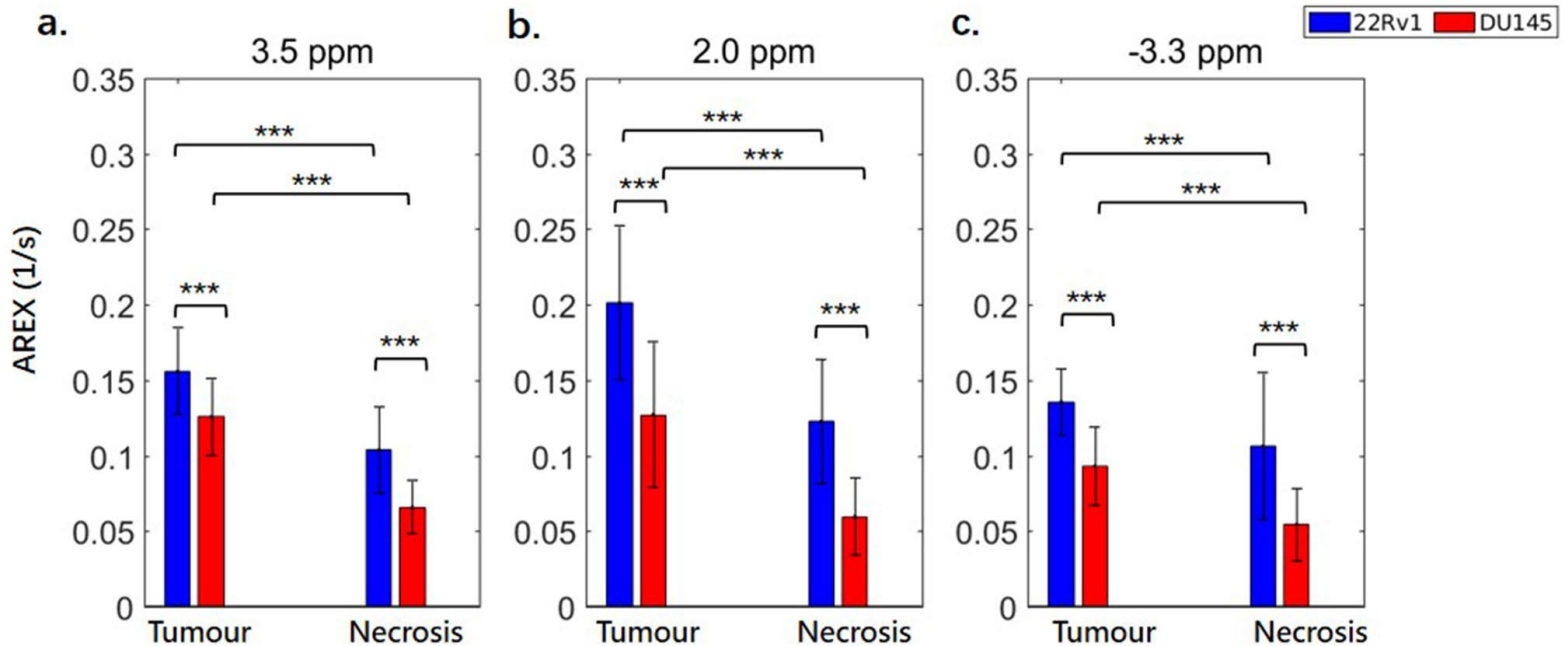
CEST and rNOE contributions of 22Rv1 and DU145 tumour and necrotic regions with a B_1_ of 2 μT. Data is shown at offsets of 3.5, 2.0, and − 3.3 ppm. There are consistent significant differences between 22Rv1 tumour and necrotic regions and between DU145 tumour and necrotic regions. The contributions are calculated using the apparent exchange rate (AREX) formula. Similar differences also exist in the comparison with a B_1_ of 0.5 μT (see Supplementary Fig. S6). The CEST and rNOE contribution calculated using conventional subtraction method have, however, failed to reveal the differences between these cell lines (see Supplementary Fig. S7). ***p < 0.001.

The CEST (3.5 and 2.0 ppm) and rNOE (− 3.3 ppm) contributions to the saturation effect at low B_1_ (0.5 and 2 μT), calculated using AREX with the extrapolated MT-only spectra as the Z-spectrum reference and minimizing the confounding effects of T_1_ at these B_1_s, are shown in Fig. 7 corresponding to the resonances of the amide (3.5 ppm), guanidinium (2.0 ppm), and aliphatic (− 3.3 ppm) groups. The spectra for MTR_REX_ and AREX are shown in the supplementary info (see Supplementary Fig. S8). The tumour regions have significantly higher (*p* < 0.001) CEST effects compared to their necrotic regions at all CEST offsets (3.5 and 2.0 ppm) in both tumour types. Both the tumour and necrotic regions of 22Rv1 tumours display significantly higher (*p* < 0.001) CEST effects at all three offsets than their counterparts in DU145 tumours.

### Histopathology

**Figure 8:**
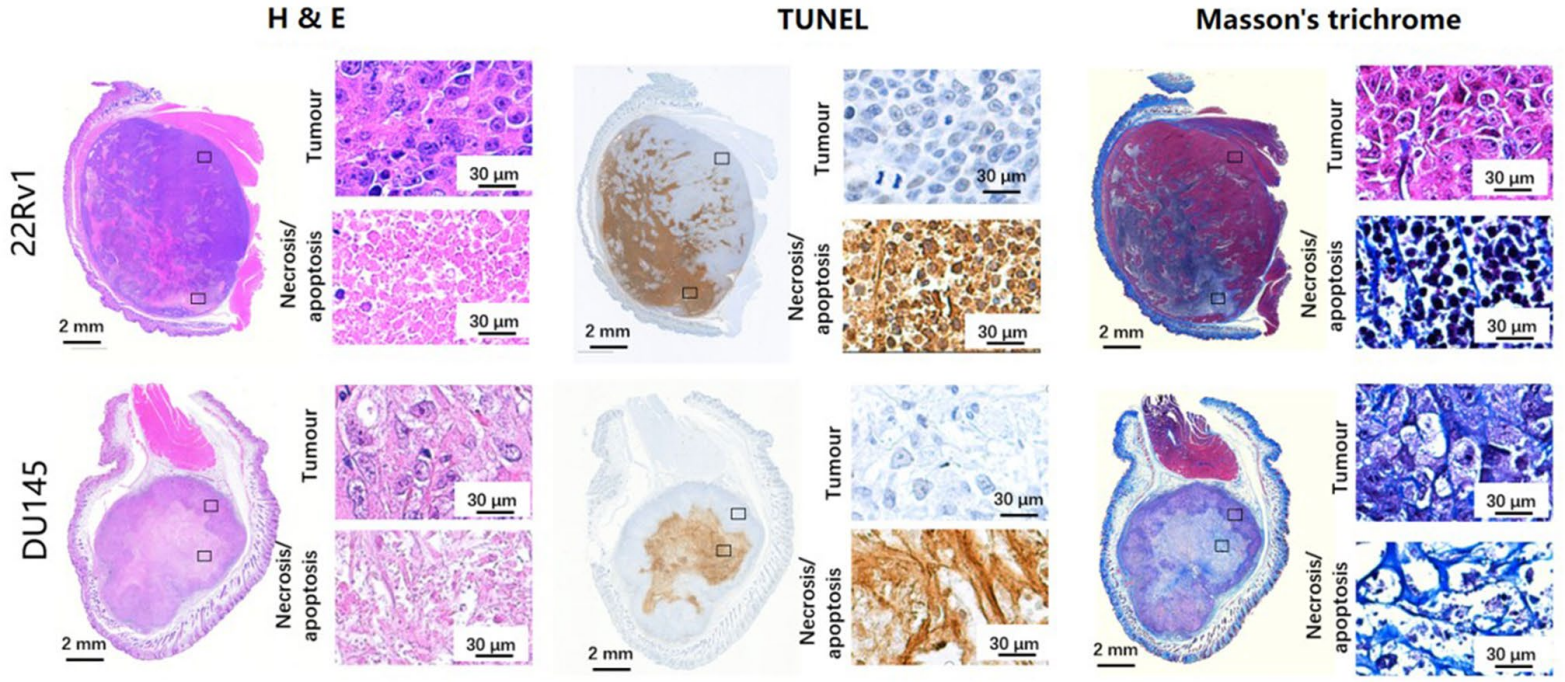
Histological stains for a representative case from each type of tumour. Whole-tumour and details of the tumour and necrotic regions for H&E, TUNEL, and Masson’s trichrome stains are shown, where both the tumour and necrotic regions in the 22Rv1 tumours display higher cell content than their counterparts in the DU145 tumours which, on the other hand, have higher connective tissue content than 22Rv1 tumours.

H&E, TUNEL, and Masson’s trichrome histopathology stains were performed on each tumour and representative cases are shown in Fig. 8. In each tumour, one zone was picked in the tumour region as well as in the necrotic region and the magnified images of these areas are also included. In the tumour regions (H&E section, Fig. 8) cells in the DU145 xenograft appear to be larger and also have a lower cellular density compared to the cells in the 22Rv1 tumour regions. The major difference between the necrotic regions (brown stain in the TUNEL assay; Fig. 8) of the two tumour types is that this region in the 22Rv1 xenograft consists primarily of apoptotic cell debris including the cell membranes and other cell contents, while that in DU145 is mostly extracellular matrix. In both tumour types, there is more connective tissue (light blue in the Masson’s trichrome stain; Fig. 8) in the necrotic regions than tumour regions. In DU145 tumours, both the tumour and necrotic regions demonstrate more connective tissue deposition throughout the tumour compared to their counterparts in the 22Rv1 tumours.

## Discussion

In this study, the saturation transfer properties of tumours derived from two PCa cell lines (22Rv1 and DU145), commonly used in translational research, were compared. DU145 was derived from a brain metastasis in a patient with metastatic castrate-resistant PCa^22^ and the 22Rv1 was a subline obtained from the CWR22 xenograft that was derived from a patient with castrate-sensitive prostate cancer^23^. The 22Rv1 subline was obtained following serial passage of xenografts in castrated mice to recapitulate the in vivo development of castrate-resistance. While there has been no previous study that compares the aggressiveness between these two types of xenografts, we have observed in our experiments that the 22Rv1 tumours display a higher growth rate compared to the DU145 tumours on average, which may suggest that the former has a higher proliferation rate, and behaves more aggressively than the latter.

Z-spectrum measurements revealed that 22Rv1 xenografts had higher saturation transfer effects than DU145 in both the tumour and necrotic regions, especially at high saturation amplitudes (B_1_s of 3 and 6 μT), where the Z-spectrum is mainly affected by MT. A two-pool model fit indicated a stronger MT effect in 22Rv1 tumours. The MT effect is defined here as the product of the exchange rate and the volume fraction, *R*_MT_*M*_0,MT_, fit using the two-pool model, and commonly reported as a product due to coupling^14^. Both the tumour and necrotic regions in the 22Rv1 tumours had significantly higher *R*_MT_*M*_0,MT_ compared to these regions in DU145 tumours. Moreover, the intrinsic transverse relaxation time of the free pool, *T*_2,F_, which is associated with mobility of free water molecules, was longer in the necrotic regions of the DU145 tumours than the 22Rv1 tumours. Histopathology showed higher cell membrane content due to higher cellular density in both the tumour and necrotic regions of 22Rv1 tumours as compared to their counterparts in DU145. The larger MT effect in the 22Rv1 xenografts could be explained by higher cellular density, leading to increased concentration of phospholipid bilayers in cells and organelle membranes, which are the major contributors to the MT effect^24^. In addition, the higher mobility of water molecules in the DU145 tumours, due to decreased cellular density, could contribute to the higher *T*_2,F_.

Shorter T_2_ relaxation times of the macromolecular pool, *T*_2,MT_, in tumour as compared with necrotic regions in both tumour types (7.8 ± 0.1 vs. 8.2 ± 0.2 µs in the 22Rv1 tumours and 7.4 ± 0.1 vs. 7.9 ± 0.4 µs in the DU145 tumours), could be related to the differences in the type of macromolecules responsible for the MT effect in these different regions. Interestingly, the necrotic regions exhibited higher MT effects than their tumour counterparts. This phenomenon could be further explained by qualitative analysis of the macromolecular content in different tumour types and regions based on histopathology.

Masson’s trichrome stain is sensitive to connective tissue with high collagen content, which is known to be a major macromolecule contributing to the MT effect^25^. Indeed, collagen-rich tissues have the highest MT effect among all tissues in the body^17^. As shown by the Masson’s trichrome stain, the necrotic regions contain more connective tissue than tumour in both tumour types. Several studies have also demonstrated the high connective tissue content of necrotic regions using Masson’s trichrome stain^26,27^, but, to our knowledge, there has been no direct comparison of the connective tissue content between different tumour types. Both the tumour and necrotic regions of the DU145 tumours had a more fibrous appearance (more collagen) on histology and lower cellular density (less lipid) than 22Rv1 tumours, but had lower MT effects than 22Rv1. Therefore, it is speculated that the higher MT effects in both of tumour and necrotic regions of 22Rv1 resulted primarily from their higher cellular density, and therefore higher lipid content, rather than collagen content. On the other hand, as shown in the TUNEL stains, the cellular density did not vary significantly between tumour and necrotic regions within the same tumour type. Therefore, it could be assumed that collagen content plays a more dominant role in contributing to the MT effects within a tumour. A previous study^28^ has pointed out that decreased collagen content in the extracellular matrix was related to increased necrotic foci and higher tumour grade, which accords with our hypothesis that 22Rv1 tumours are more aggressive due to their high growth rate. This study also revealed that high collagen synthesis was often noticed in the host tissue around the tumour to serve as a barrier to impede tumour invasion, which could explain the high MT effect in the muscle region shown in Fig. 3.

To evaluate the CEST contribution, we used a modification of the AREX metric developed by Windschuh et al.^29^. The original multi-pool AREX method requires the acquisition of the entire Z-spectrum with low B_1_, which is time consuming, and typically assumes two CEST pools and one rNOE pool to calculate the contribution from each pool. The adapted method relies on the acquisition of Z-spectra with high B_1_ and a T_1,obs_ map to extrapolate the MT reference and acquisition of Z-spectrum point(s) with low B_1_ only at offsets of interest ^21^. This is faster, but means that the AREX values calculated in this paper potentially contain contributions from the overlapping spectral contributions of multiple chemical groups. When using relatively low B_1_ amplitudes, such as 0.5 µT, the labeling will not be perfectly efficient due to influences from the solute exchange rate and T_2_, which varies for each of the CEST and NOE pools. In our experiments, AREX is not independent from B_1_ due to labeling efficiency being less than unity. However, comparisons between the two tumour types for the AREX metric at any given B_1_ and solute pool are expected to be largely unaffected by variation in labeling efficiency. Calculations using the B_1_ used in this study and the solute exchange rates and T_2_s found in literature^30^, show that a 10% increase in either the solute exchange rate or T_2_ of the three pools considered affects the efficiency by 0.03 or less and a 20% increase affects efficiency by 0.06 or less.

For both tumour types, the necrotic regions had a significantly lower CEST effect compared to tumour, which is consistent with necrotic regions having decreased metabolism. The tumour regions in 22Rv1 tumours displayed significantly higher CEST effects compared to those of the DU145 tumours. This can be explained by the higher cellular density of the 22Rv1 yielding a higher metabolite concentration and therefore stronger CEST effects. Although in this study the effect of extracellular/intracellular pH on CEST was not investigated, previous studies have pointed out that increased extracellular pH could lead to hyperintensity of amide signal (3.5 ppm)^31^ and the increased intracellular pH has been correlated with increased amide exchange rate in glioblastoma tissue^32^. NOE has also been proved to be pH insensitive^33^.

In this study, saturation transfer properties showed great potential in assessing solid tumours by providing information relating to intratumoural microstructure including cellular density, cell membrane integrity, and intratumoural tissue composition, which have been related to tumour aggressiveness and prognosis^34,35^. Furthermore, the intratumoural metabolic properties identified using CEST could guide tumour treatment^36,37^. This study, for the first time, analyzed and compared the saturation transfer properties between two types of prostate tumours, which are speculated to have different aggressiveness. We discovered that the 22Rv1 tumours which are potentially more aggressive are characterized with higher MT (higher cellularity) and higher CEST effects (higher metabolism) than DU145 tumours. This comparison provided important pioneering references for future preclinical studies in identifying the stage and malignancy of solid tumours with a non-invasive modality.

## Methods

### Animal model

Two prostate adenocarcinoma cell lines were used in this study: DU145^22^ and 22Rv1^23^ (ATCC, Manassas, VA). DU145 originates from a brain lesion of metastatic PCa in a patient. 22Rv1 is derived from a serially propagated xenograft CWR22R developed from a parental human PCa xenograft CWR22. For each cell line, approximately 3 × 10^6^ cells mixed in a 1:1 ratio by volume with growth factor reduced Matrigel matrix (BD Canada, Mississauga, ON) were injected in the right hind limbs of female athymic nude mice (*n*_DU145_ = 34, *n*_22Rv1_ = 32; Charles River Canada, Saint-Constant, QC) and allowed to grow into tumours for at least 34 days post-injection. Tumours were measured using callipers every 1–4 days and their volume was calculated using the formula volume = length × width^2^/2. All experimental procedures were approved by the Animal Care Committee of the Sunnybrook Research Institute, which adheres to the Policies and Guidelines of the Canadian Council on Animal Care and meets all the requirements of the Animals for Research Act of Ontario and the Health of Animals Act of Canada.

### Magnetic resonance imaging

Tumours were scanned at 7 T (BioSpec 70/30 USR with B-GA12S HP gradients running ParaVision 6.0.1, Bruker BioSpin, Billerica, MA) using an 86 mm inner diameter volume coil for transmit and a 20 mm diameter loop surface coil for receive. A fifteen-slice 2D axial T_2_-weighted rapid acquisition with refocused echoes^38^ scan (RARE; TR = 2500 ms; TE_eff_ = 55 ms; FOV = 20 mm × 20 mm; slice thickness = 0.5 mm; matrix = 128 × 128; RARE factor = 12; bandwidth = 33 kHz; averages = 4; 6 min, 40 s) was used for prescribing the slice of interest, chosen to be at the thickest point of the tumour. B_0_-map-based shimming (MapShim) of second order gradients was performed on an ellipsoidal volume enclosing the tumour in the slice of interest. Flip angle scale factor maps^39^ were calculated for the first four mice using a series of 3D high flip angle fast low angle shot (FLASH)^40^ scans and the T_1_ map for the slice of interest. The flip angle in the tumour region of interest was found to be within 6% of nominal. Thus, B_1_ correction was deemed unnecessary going forward.

For the DU145 tumours, saturation transfer-weighted images were acquired using one 490 ms block RF saturation pulse per k-space line and single-slice FLASH acquisition (TR = 500 ms; TE = 3 ms; flip angle = 30°; FOV = 20 mm × 20 mm; slice thickness = 1 mm; matrix = 64 × 64; bandwidth = 50 kHz; dummy scans = 1) as in our previous work^41^. For the 22Rv1 tumours, saturation transfer-weighted images were acquired using one 4900 ms block RF saturation pulse and four-shot, centrically encoded, single-slice RARE acquisition (TR = 5000 ms; TE_eff_ = 4.75 ms; flip angle = 90°; same FOV and matrix as FLASH; RARE factor = 16; bandwidth = 50 kHz; dummy scans = 1), which produced Z-spectra identical to those from the FLASH sequence (see Supplementary Fig. S6), but with a much shorter acquisition time. The cumulative saturation time when acquiring the centre of k-space was approximately 16 s for RARE and 10 s for FLASH, which was sufficient for the system to reach steady-state saturation. The use of four-shot RARE instead of FLASH reduced the acquisition time from 32 to 20 s per frequency offset.

To allow for correction of system instability in post-processing, reference scans at Δω = 667 ppm were acquired before and after and also interleaved between every five Z-spectrum measurements^41,42^. For the DU145 tumours, the scan time for the Z-spectra including reference scans with B_1_ = 0.5 and 2 µT was 44 min/spectrum; 3 and 6 µT, 8.5 min/spectrum; and 0.1 µT, 15 min. For the 22Rv1 tumours, the scan time was shortened to 28 min/spectrum, 5.5 min/spectrum, and 9.5 min, respectively.

To evaluate the longitudinal relaxation time T_1_, five inversion recovery RARE scans (TR = 10,000 ms; TE_eff_ = 10 ms; TI = 30, 110, 390, 1400, 5000 ms; same FOV and matrix as FLASH; RARE factor = 4; bandwidth = 77 kHz; 2 min each) were also acquired for a T_1_ map^43^. The T_2_ maps were calculated using a T_1_ map and WASSR. The total acquisition time including scout and shimming was 2–2.5 h per animal.

### Histopathology

Tumours were excised for histopathological assessment immediately after scanning. Each tumour was isolated and marked with a suture on the proximal margin for subsequent alignment with MRI, formalin fixed for 24–48 h, and then stored in 70% ethanol. Tumours were trimmed for sectioning in the region that corresponded as closely as possible to the MRI slice. Tissues were paraffin embedded, sectioned at 10 µm, and mounted on slides. Three types of histological section were prepared: H&E staining for structural detail, a TUNEL assay using 3,3′-diaminobenzidine (DAB) chromogen and haematoxylin counter staining for necrosis, and a Masson’s trichrome stain for distinguishing connective tissue content. The tissue section that best correlated with the MRI slice was imaged using an Axio Imager 2 (version M2, Carl Zeiss Canada Ltd., Toronto, ON)^44^ microscope with the Stereo Investigator (MBF Bioscience, Williston, VT) stereology system at magnification 20 × and 60 × for details^42^.

### MRI data pre-processing

For each animal, images were registered using a rigid body transformation to the first reference image acquired with B_1_ = 0.5 µT. In order to avoid misregistration of low SNR images acquired with saturation near the water resonance, Z-spectrum images with less than 50% of the mean signal of the reference scan were registered using the transformation matrix of the last image with sufficient SNR, typically an interleaved reference scan. Baseline drift correction of all Z-spectrum scans consisted of fitting a straight line to the interleaved reference scans. This was followed by spectrum-wise B_0_ correction of the WASSR and Z-spectrum images with low B_1_ (0.5 and 2 µT). The correction consisted of fitting one Lorentzian (corresponding to the DE contribution) to the WASSR Z-spectrum at frequency offsets between ± 0.5 ppm and a sum of two Lorentzians (corresponding to the DE and MT contributions) to the low B_1_ Z-spectra. The spectra were re-centred to the peak position of the DE Lorentzian and linearly interpolated. High B_1_ images (which were largely MT sensitive) were acquired with logarithmically spaced offsets ranging from 3 to 300 ppm. Thus, B_0_ correction was not required for these spectra.

The T_2_ maps were calculated using a T_1_ map and WASSR. First, the T_1_ map (*T*_1,obs_) was calculated from the inversion recovery scans by fitting to the inversion recovery RARE signal equation^43^. Then, a T_2_ map (*T*_2,obs_) was calculated from the T_1_ map and WASSR Z-spectrum using the steady-state direct water saturation signal intensity as in previous work^42^:

1 $$S\left(\Delta \omega \right)={S}_{0}\frac{{R}_{1}\left[{R}_{2}^{2}+{\left\{\Delta \omega \right\}}^{2}\right]}{{R}_{1}\left[{R}_{2}^{2}+{\left\{\Delta \omega \right\}}^{2}\right]+{\omega }_{1}^{2}{R}_{2}},$$

where *R*_1_ = 1/*T*_1,obs_, *R*_2_ = 1/*T*_2,obs_, and ω_1_ = γ*B*_1_. T_1_ and T_2_ values were normalized by 4000 and 300 ms, respectively, which were values selected as being slightly higher than the highest values typically seen in tumour regions to match the range of the saturation transfer images prior to segmentation.

The pre-processing above was performed in MATLAB (Release 2018b, The MathWorks, Natick, MA)^45^. Subsequent processing was performed in Python (version 3.7)^46^ with the SciPy (version 1.2.1)^47^ scientific computing, OpenCV (version 3.4.1)^48^ computer vision, and scikit-learn (version 0.20.3)^49^ machine learning libraries.

Image erosion was used to remove edge voxels, which can be contaminated by partial volume effects. Voxels with a B_0_ shift of greater than 0.5 ppm were excluded, so only well-shimmed voxels were used. Erosion was performed using the *binary_erosion* function in SciPy using a rank 2 structuring element where all elements are neighbours.

### Automatic segmentation

**Figure 9:**
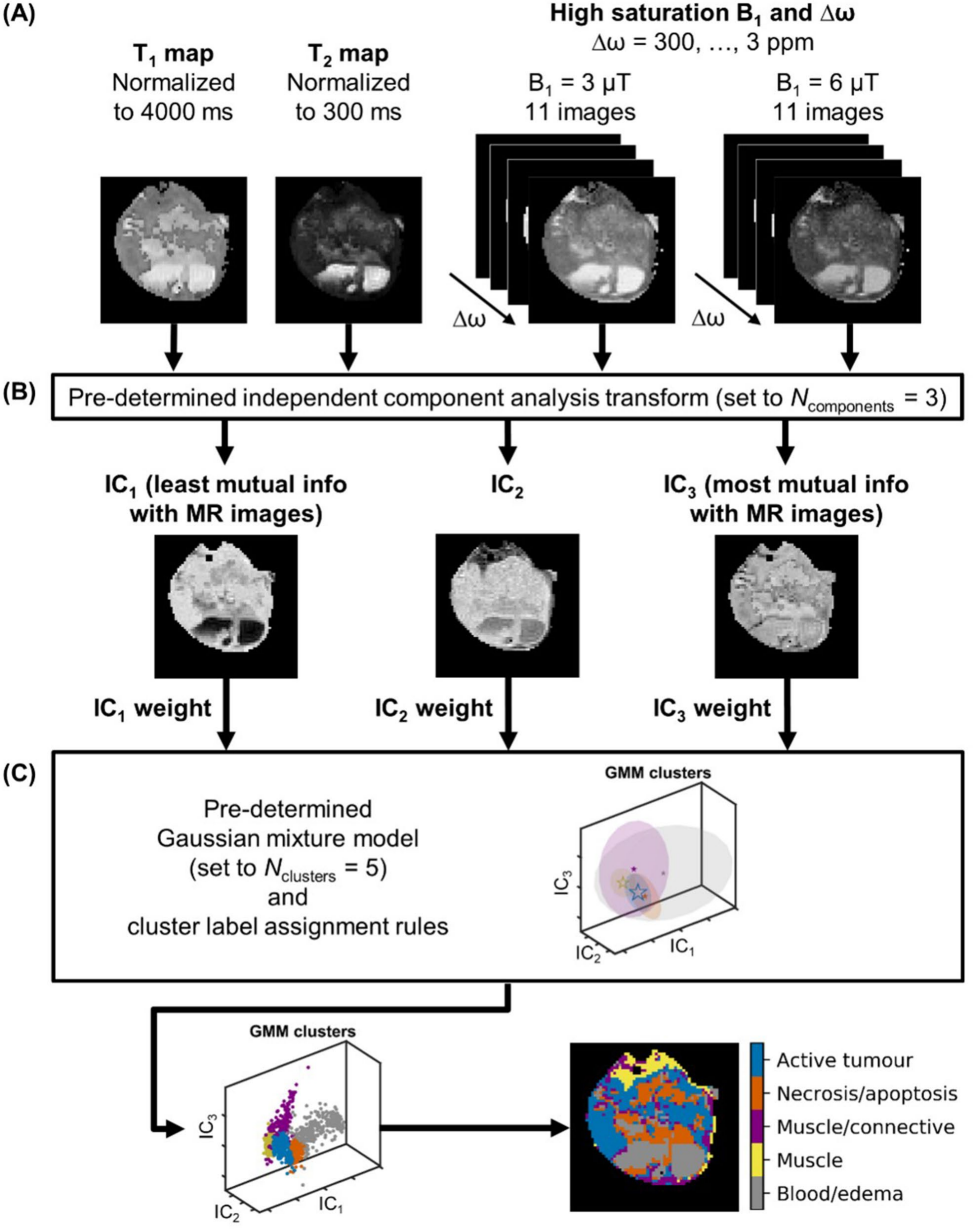
The automatic segmentation pipeline. (**a**) Normalized T_1_ and T_2_ maps and Z-spectrum images acquired with various saturation B_1_ amplitudes and at various frequency offsets Δω (3 ppm shown) for an illustrative DU145 mouse. (**b**) Non-background voxels were concatenated into an observation matrix and transformed by a trained independent component analysis transform set to generate three independent component (ICs). As part of training, the ICs were sorted in order of increasing mutual information with respect to the input and each IC was weighted. A different set of IC weights was used for each of the two tumour types. (**c**) The ICs were then input to the Gaussian mixture model set to five clusters.

The segmentation pipeline, developed in our lab^21^, is shown in Fig. 9 and used T_1_ and T_2_ maps and Z-spectrum images acquired at high B_1_ (3 and 6 µT) as input (Fig. 9a). These were concatenated to generate an observation matrix for each tumour type. For each observation matrix, an independent component analysis (ICA) was performed using the FastICA algorithm^50^. ICA is a linear transformation from the original feature space to a new one such that the new features are mutually independent (Fig. 9b). Transformation into three independent components (ICs) was chosen based on our previous work^21^. In this study, the ICs of each dataset were sorted in order of increasing mutual information between each component and the average of all protocol images, calculated using the *normalized_mutal_info_score* function in scikit-learn normalized to the arithmetic mean of the ICs and average images, and labelled IC_1_, IC_2_, and IC_3_. The ICs were then weighted (IC_1_:IC_2_:IC_3_ weightings of 2:3:1 were used for 22Rv1 images and 1:3:2 for DU145, which resulted in segmentation masks that visually best matched histology) and input to a Gaussian mixture model (GMM)^51^, which is a probabilistic model that identifies clusters with Gaussian distributions within IC space (Fig. 9c). For each GMM cluster, the fitting estimated a weighting, along with a mean in the three-dimensional IC space, and a full covariance matrix (i.e., each Gaussian may adopt any position and shape). Based on our previous study, the optimal number clusters was five. All clusters were associated with histology results and assigned to active tumour, necrosis/apoptosis, muscle, muscle/connective tissue and blood/edema.

After GMM fitting, the following label assignment algorithm was applied to the DU145 segmentation masks as in our previous work^21^: (1) the cluster with the largest absolute value of the GMM mean of IC_1_ was labelled blood/edema; (2) each dataset was reflected about the IC_1_ = 0, IC_2_ = 0, and IC_3_ = 0 planes, as required, such that the blood/edema cluster was in the first octant since ICA does not identify the sign of the source signals; (3) of the remaining clusters, the one with the smallest (i.e., most negative) GMM mean of IC_2_ was labelled muscle; the second smallest, muscle/connective; the second largest, necrosis/apoptosis; and the largest, active tumour. A similar algorithm was applied to the 22Rv1 masks except that the clusters in step 3 were assigned in the following order: active tumour, muscle/connective, necrosis/apoptosis, and muscle. The segmentation results were visually connected to histology stains (H&E and TUNEL).

### Quantitative MT model fitting

T_1_ maps and Z-spectra with B_1_ = 0.1, 3, and 6 µT were fitted to a two-pool MT model using a super-Lorentzian lineshape for the semisolid macromolecular pool for the tumour and necrosis/apoptosis voxels^52^. The four free parameters are the T_2_ of the free pool (*T*_2,F_), the exchange rate of magnetization from the MT to the free pool (*R*_MT_), the equilibrium magnetization of the MT pool relative to the free pool (*M*_0,MT_), and the T_2_ of the MT pool (*T*_2,MT_). Since the parameters *R*_MT_ and *M*_0,MT_ are coupled, their product was used for further analysis and termed the MT effect. All parameters were fitted for the tumour and necrosis/apoptosis regions of individual mice and then averaged together. The difference between the MT effect between tumour and necrosis/apoptosis regions over all mice were compared using unpaired Student’s t-tests.

### Isolation of CEST and rNOE contributions

Since Z-spectra are sensitive to direct water saturation, MT, CEST, and rNOE at different offset ranges, it is necessary to isolate each of them to reduce confounds. Based on the method introduced by Heo et al.^53^, the extrapolated semi-solid magnetization transfer reference (EMR) was calculated using the MT model parameters, which represents the MT effect. Adapting the technique described by Windschuh et al.^29^, the T_1_-corrected apparent exchange-dependent relaxation (AREX) metric for CEST and rNOE contributions from each tumour and necrosis/apoptosis regions was calculated as follows:

2 $${\text{MTR}}_{\text{REX}}=\frac{1}{{Z}_{\text{lab}}}-\frac{1}{{Z}_{\text{EMR}}}$$

3 $${\text{AREX}}=\frac{{\text{MTR}}_{\text{REX}}}{{T}_{1,F}}$$

where the measured Z-spectrum (B_1_s of 0.5 and 2 µT were each used) is denoted *Z*_lab_, the extrapolated MT reference is *Z*_EMR_, and *T*_1,obs_ is the measured T_1_. The difference between the mean CEST-only contribution at 3.5 (amide CEST), 2.0 (guanidinium CEST), and − 3.3 ppm (aliphatic rNOE) between tumour and necrosis/apoptosis regions over all mice were compared using unpaired Student’s t-tests.

## Supplementary Information

Supplementary Information

## Data Availability

The data that support the findings of this study are available from the corresponding author upon reasonable request.

## Code availability

The code used this study is available from the corresponding author upon reasonable request.
