# Supplementary material for: Saturation transfer properties of tumour xenografts derived from prostate cancer cell lines 22Rv1 and DU145
Source: Sci Rep. 2020 Dec 4;10:21315. doi: 10.1038/s41598-020-78353-8 (PMC7718243; doi:10.1038/s41598-020-78353-8)
Supplement: Supplementary file 1 — Supplementary Information [file 41598_2020_78353_MOESM1_ESM.pdf]

Saturation Transfer Properties of Tumour Xenografts Derived from Prostate Cancer Cell Lines 22Rv1 and DU145

Supplementary Information

Ziyu Tan, Wilfred W. Lam, Wendy Oakden, Leedan Murray, Margaret M. Koletar, Stanley K. Liu, Greg J. Stanisz

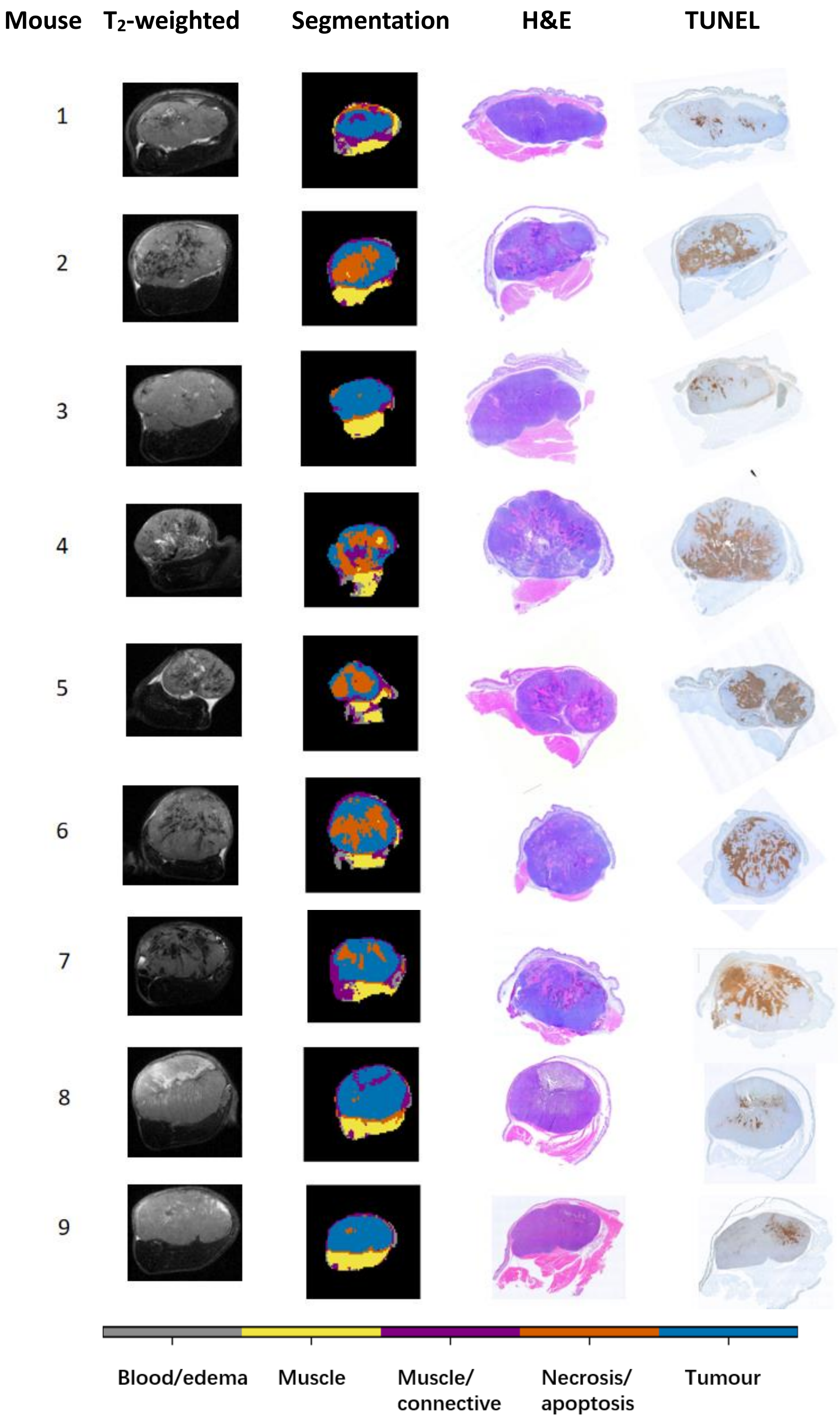

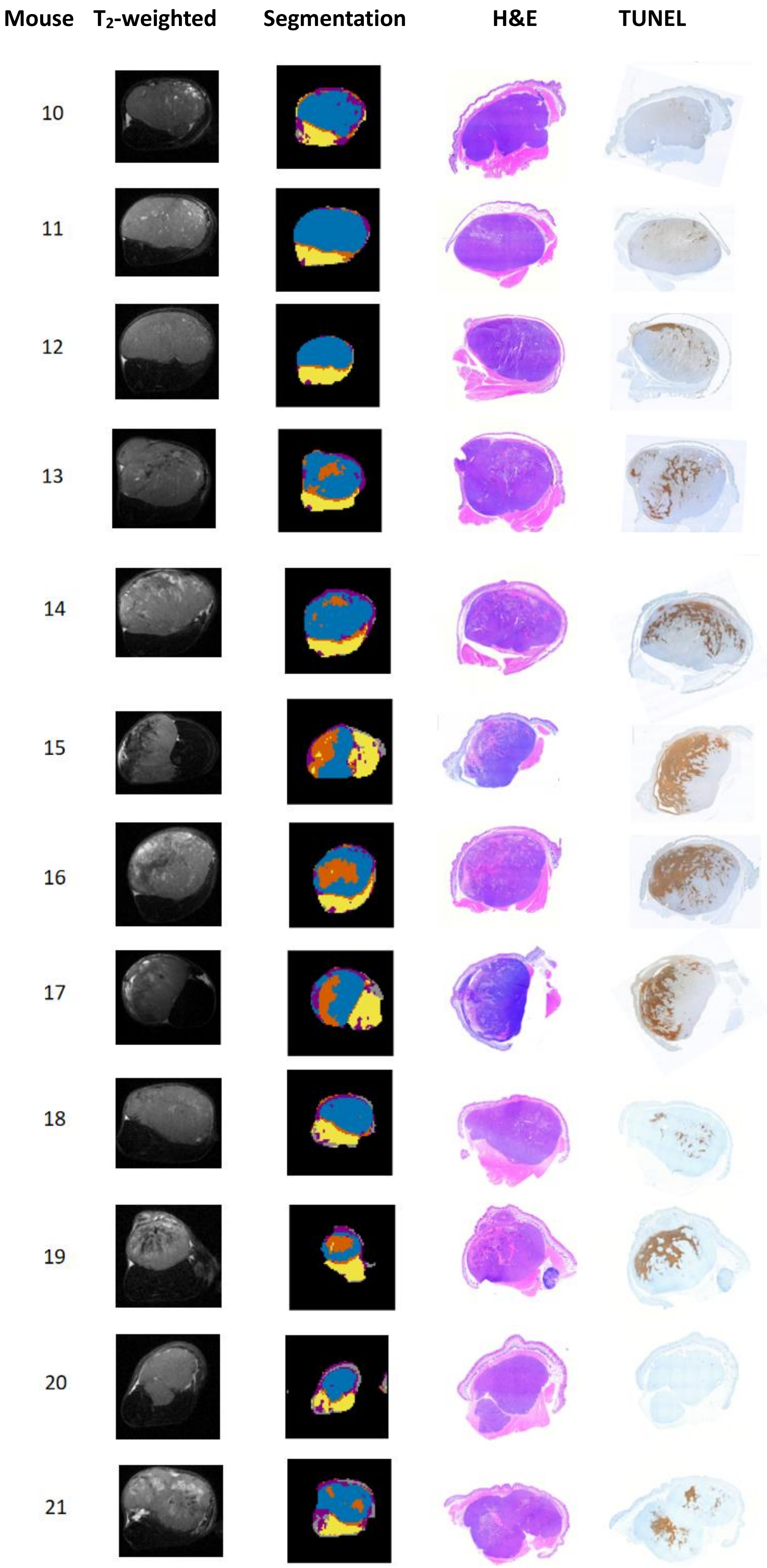
Mouse
T$_2$-weighted
Segmentation
H&E
TUNEL
10
11
12
13
14
15
16
17
18
19
20
21

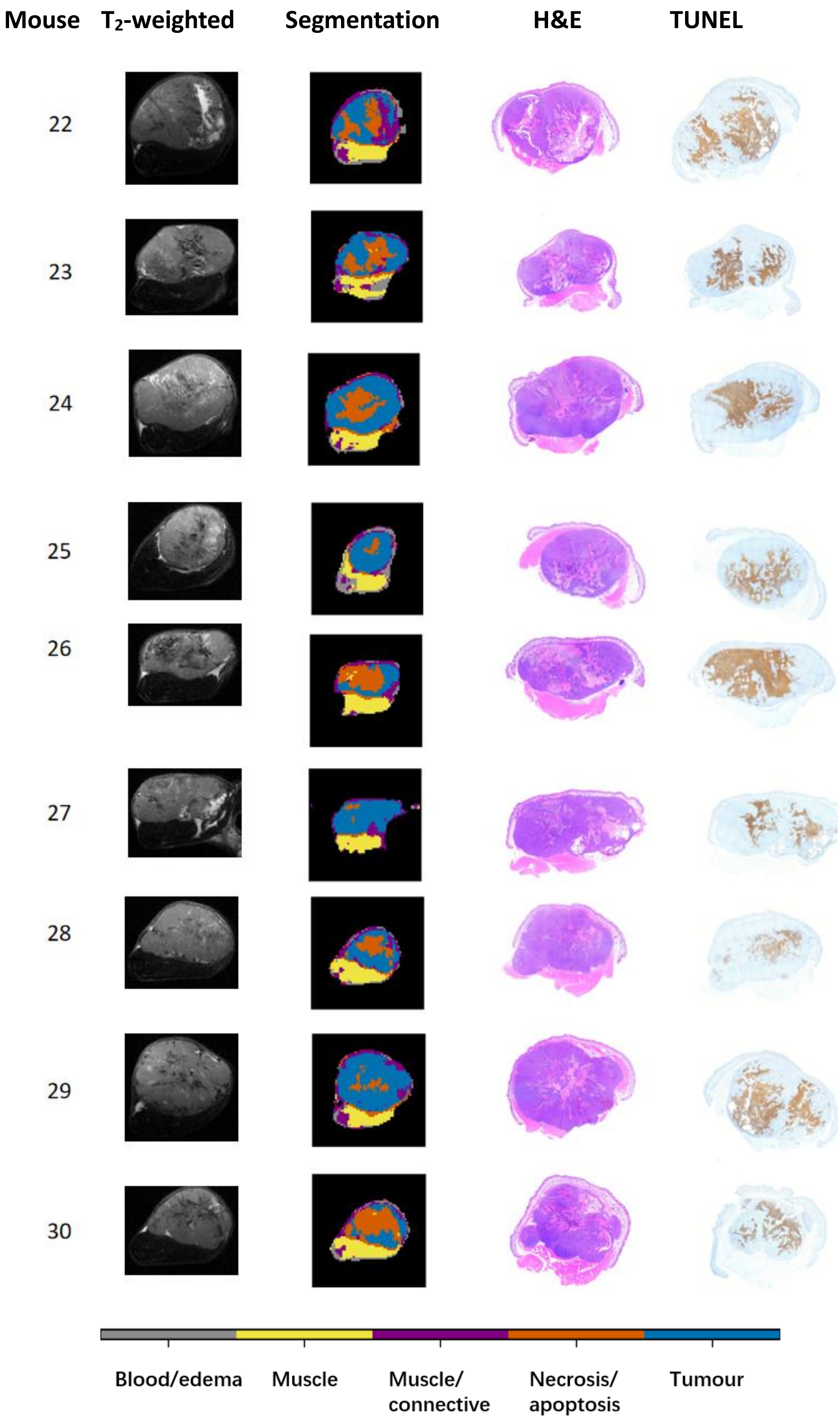

Fig. S1 The segmentation results of 22Rv1 tumours with T_2 image and histology references. The 22Rv1 tumours (n = 30) were segmented using IC_1, IC_2, and IC_3 weights of 2, 3, and 1, respectively, into 5 clusters, which are associated with the histology images and identified as active tumour, muscle/connective, necrosis/apoptosis, and muscle.

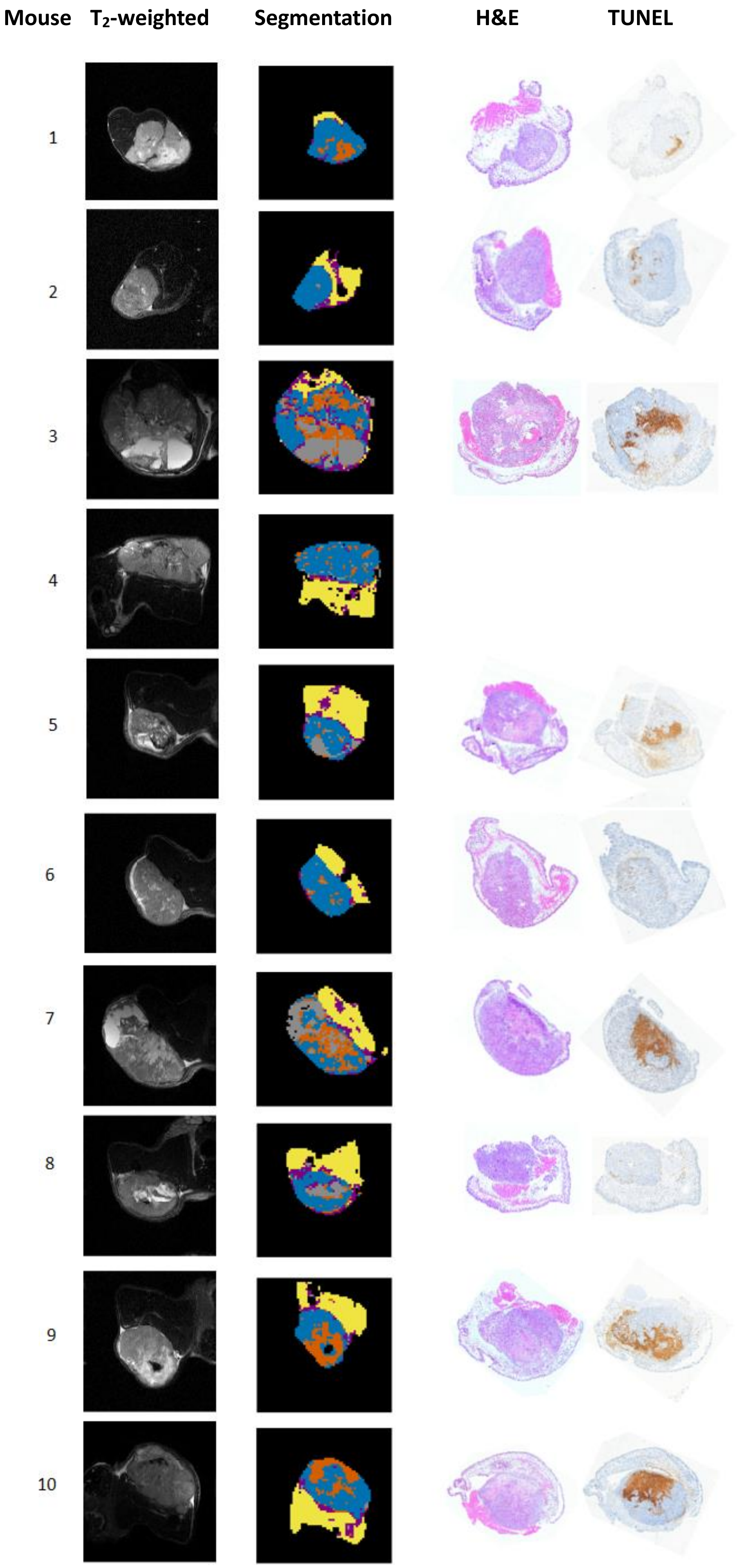
Mouse
T2-weighted
Segmentation
H&E
TUNEL
1
2
3
4
5
6
7
8
9
10

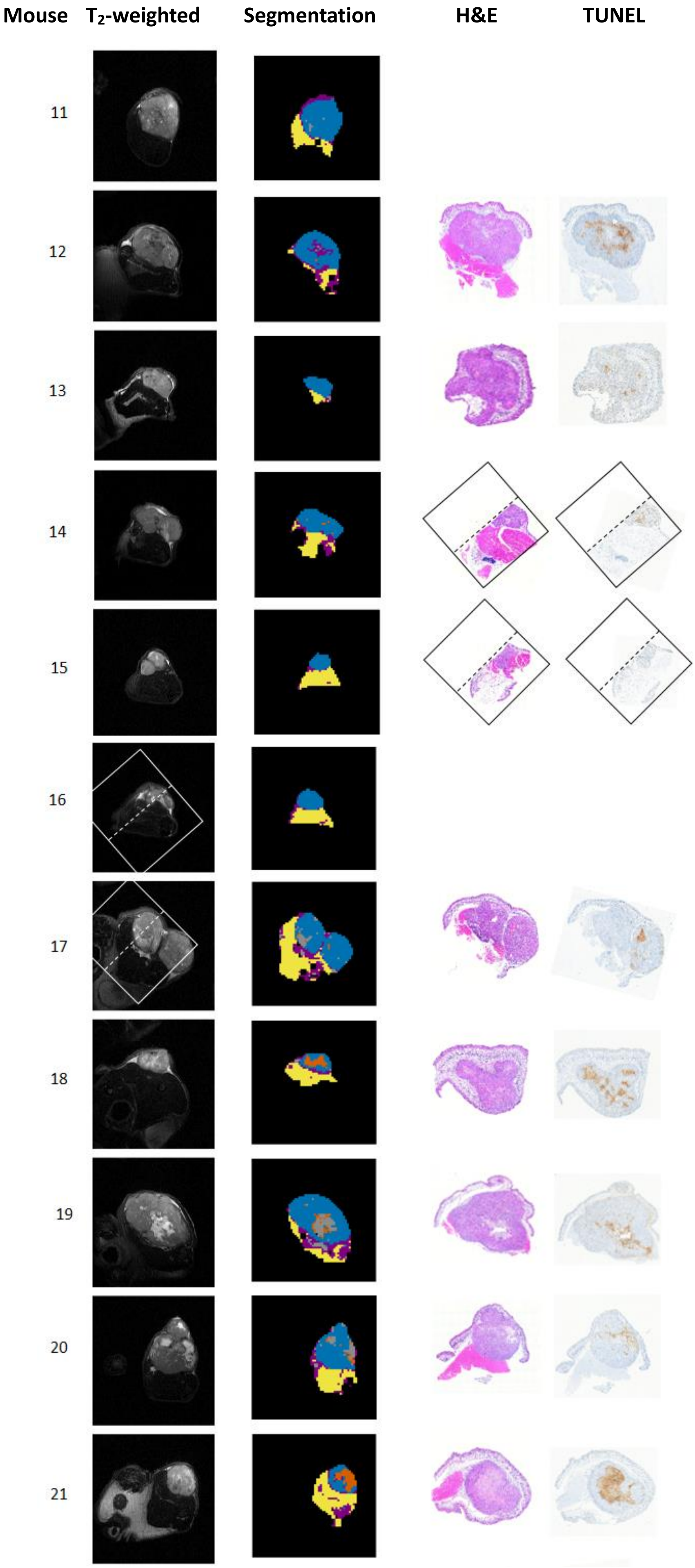

Mouse
T2-weighted
Segmentation
H&E
TUNEL
11
12
13
14
15
16
17
18
19
20
21

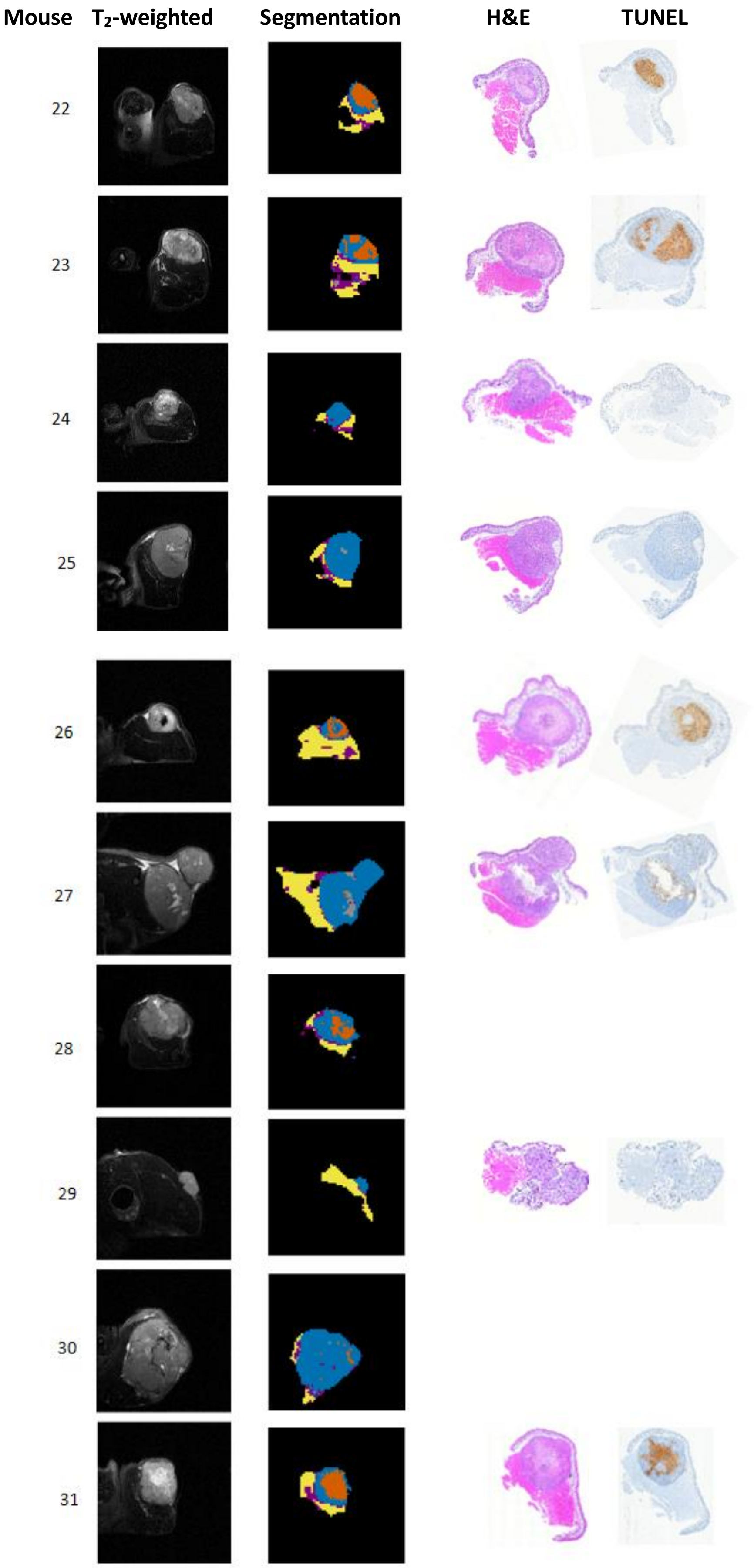
Mouse
T2-weighted
Segmentation
H&E
TUNEL
22
23
24
25
26
27
28
29
30
31

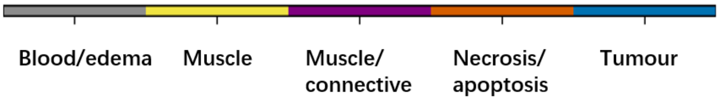
Blood/edema
Muscle
Muscle/ connective
Necrosis/ apoptosis
Tumour

Fig. S2 The segmentation results of DU145 tumours with T_2 image and histology references. The DU145 tumours (n = 31) were segmented using IC_1, IC_2, and IC_3 weights of 1, 3, and 2, respectively, into five clusters, which are associated with the histology images and identified as active tumour, muscle/connective, necrosis/apoptosis, and muscle.

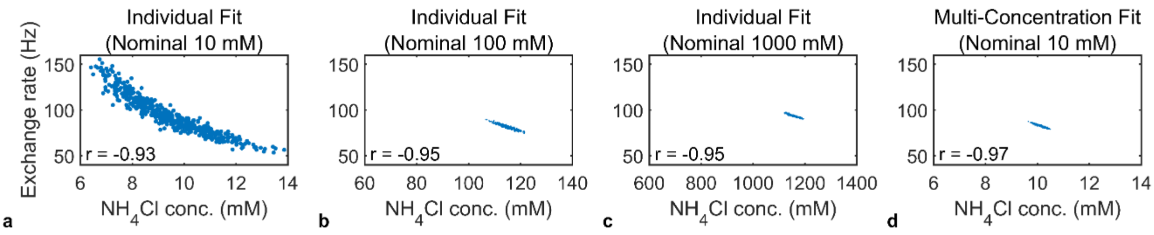

Fig. S3 Representative plots of exchange rate (R_{MT}) vs. concentration of the solute pool (proportional to $M_{0,MT}$). R_{MT} is negatively correlated with $M_{0,MT}$ in (a–c) various single- solute-concentration dataset fittings and (d) simultaneous fitting of the three datasets in a–c. (Ref. Lam WW, Oglesby RT, Stanisz GJ. Can solute exchange rate be decoupled from concentration with measurements over a range of concentrations? *Music City CEST*, Nashville, 2017.)

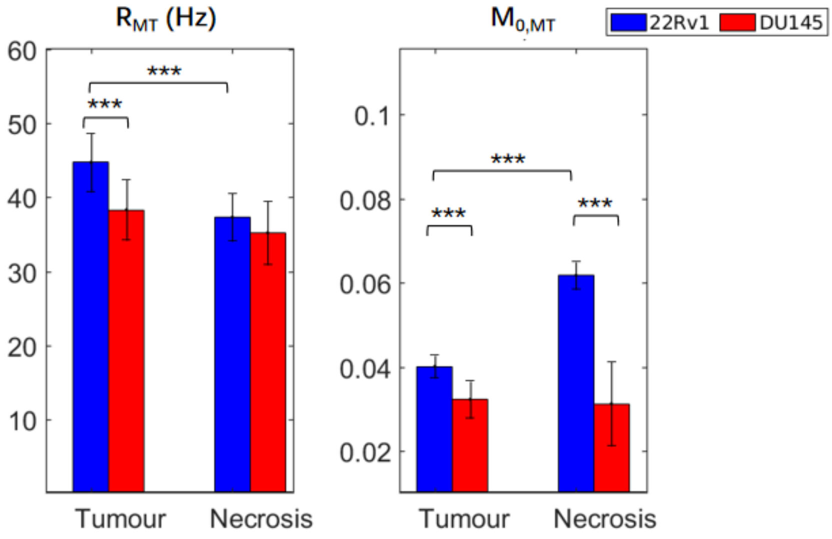

Fig. S4 Comparisons of the exchange rate of magnetization from the MT to the free pool (R_{MT}) and equilibrium magnetization of the MT pool relative to the free pool ($M_{0,MT}$) between the two tumour types and tumour and necrosis regions. **p<0.01. ***p < 0.001.

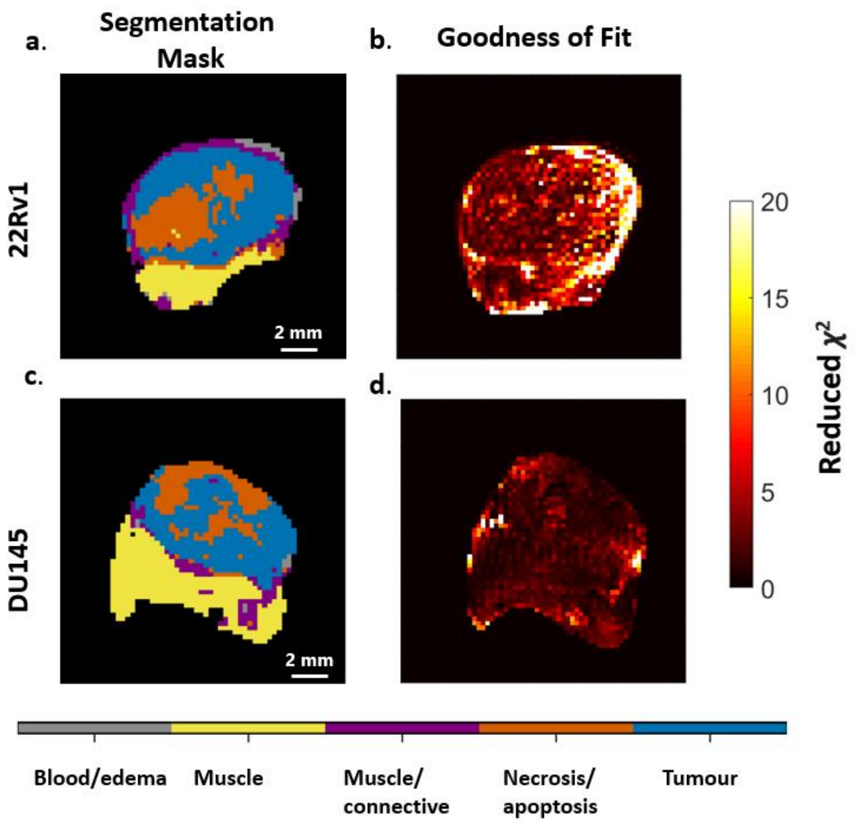

Fig. S5 (a, c) Segmentation masks and (b, d) reduced χ^2 goodness of fit maps of the representative tumours in Fig. 3.

Table S1 The reduced χ^2 goodness of fit metric of 22Rv1 and DU145 tumour and necrotic regions.

	**22RV1**	**DU145**
**Tumour**	4 ± 3	6 ± 5
**Necrosis**	3 ± 2	7 ± 7

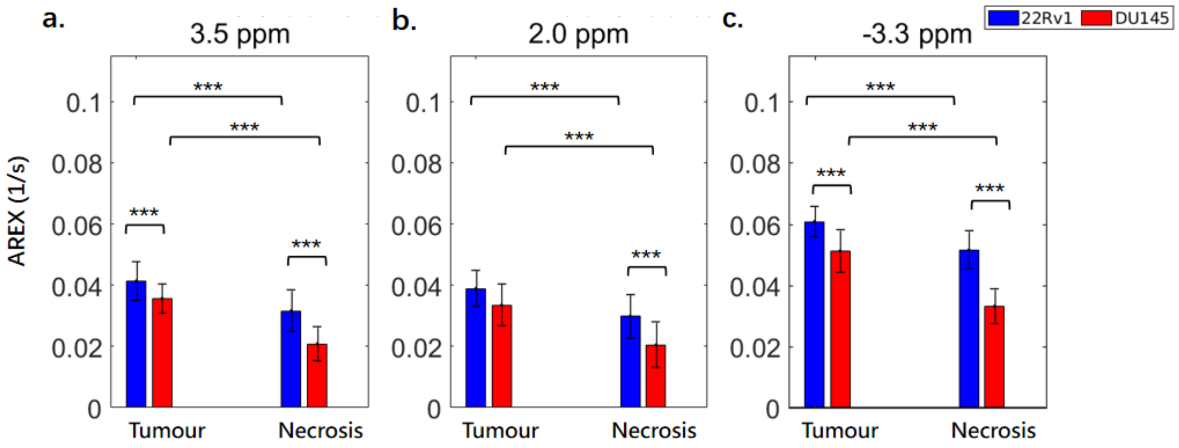

Fig. S6 CEST and rNOE contributions of 22Rv1 and DU145 tumour and necrotic regions for a B_1 of 0.5 µT. Data is shown at offsets of 3.5, 2.0, and -3.3 ppm. There are consistent significant differences between 22Rv1 tumour and necrotic regions and between DU145 tumour and necrotic regions except between the tumour regions of 22Rv1 and DU145 at 2.0 ppm. The differences between 22Rv1 and DU145 tumours are mostly consistent with the ones at B_1 of 2 µT. ***p*<0.01. ****p* < 0.001.

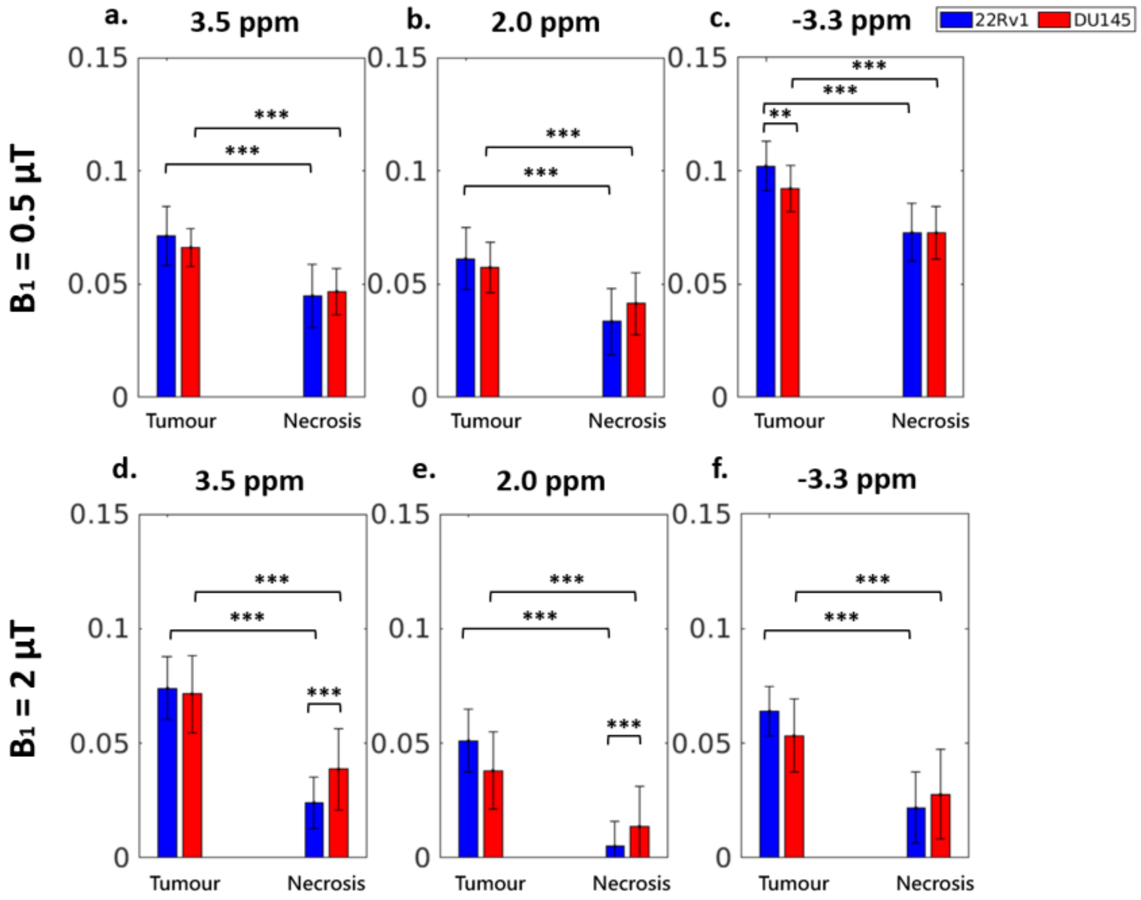

Fig. S7 CEST and rNOE contributions of 22Rv1 and DU145 tumour and necrotic regions calculated using conventional subtraction method of B1 at 0.5 µT (a- c) and 2 µT (e- f). Compared to the AREX method, the conventional method also shows similar differences between tumour and necrotic regions but failed to reveal the differences between these two cell lines. ***p*<0.01. ****p* < 0.001.

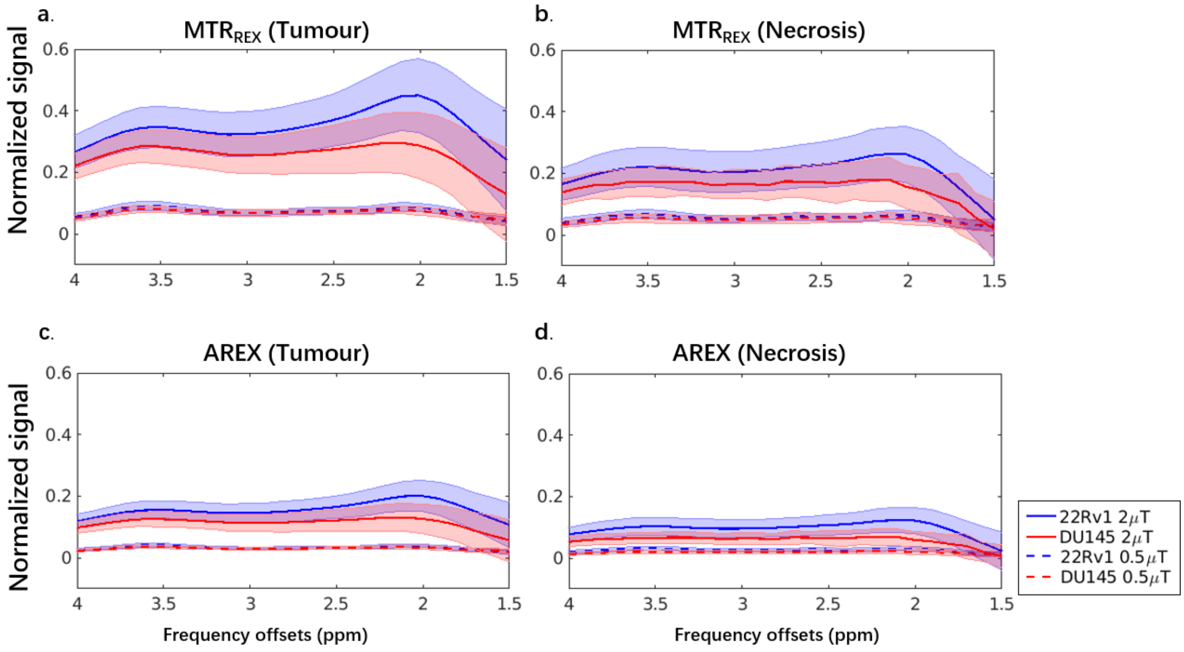

Fig. S8 The MTR_{REX} (without T_1 correction) and AREX (with T_1 correction) spectra of 22Rv1 and DU145 tumours with B_1s of 0.5 µT and 2 µT. The difference between these two types of tumours are more noticeable with a B_1 of 2 µT.

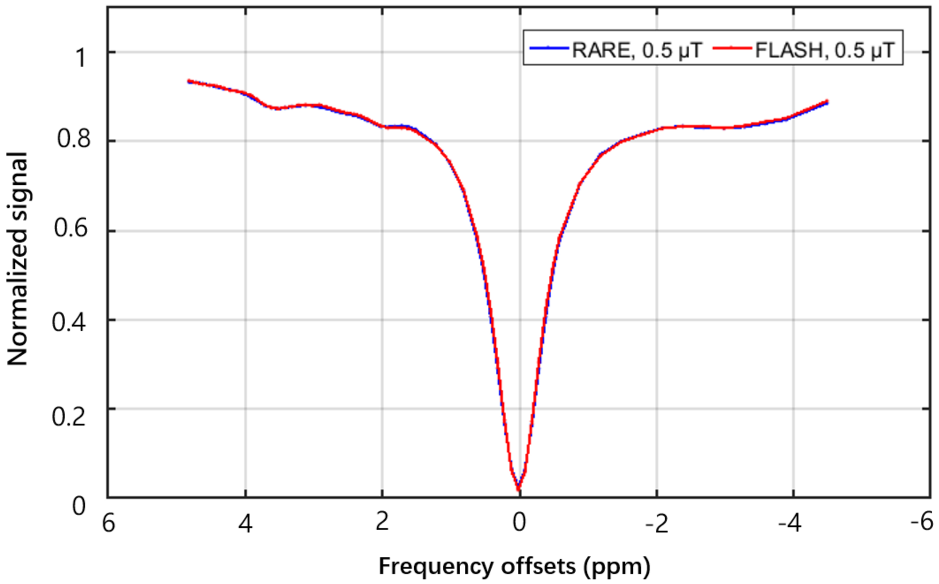

Fig. S9 Z-spectra collected using RARE and FLASH sequences at B_1 = 0.5 µT. The Z-spectra were acquired on one mouse tumour xenograft to test the comparability between RARE (TR = 5000 ms; TE = 4.75 ms; flip angle = 90°; FOV = 20 mm × 20 mm × 1 mm; matrix = 64 × 64; bandwidth = 50 kHz; and 1 dummy scan) and FLASH (TR = 500 ms; TE = 3 ms; flip angle = 30°; FOV = 20 mm × 20 mm × 1 mm; matrix = 64 × 64; bandwidth = 50 kHz; and 1 dummy scan) sequences.
